# Versatile Applications of Metal Single‐Atom @ 2D Material Nanoplatforms

**DOI:** 10.1002/advs.201901787

**Published:** 2019-08-27

**Authors:** Bin Zhang, Taojian Fan, Ni Xie, Guohui Nie, Han Zhang

**Affiliations:** ^1^ SZU‐NUS Collaborative Innovation Center for Optoelectronic Science & Technology International Collaborative Laboratory of 2D Materials for Optoelectronics Science and Technology of Ministry of Education College of Physics and Optoelectronic Engineering Shenzhen University Shenzhen 518060 China; ^2^ Institute of Translation Medicine Shenzhen Second People's Hospital First Affiliated Hospital of Shenzhen University Shenzhen 518035 China

**Keywords:** 2D materials, chemicals, energy, environment, single‐atom catalysts

## Abstract

Recently, emerging 2D material‐supported metal single‐atom catalysts (SACs) are receiving enormous attention in heterogeneous catalysis. Due to their well‐defined, precisely located metal centers, unique metal–support interaction and identical coordination environment, these catalysts serve as excellent models for understanding the fundamental issues in catalysis as well as exhibiting intriguing practical applications. Understanding the correlations between metal–support combinations and the catalytic performance at the atomic level can be achieved on the SACs@2D materials nanoplatforms. Herein, recent advances of metal SACs on various types of 2D materials are reviewed, especially their exciting applications in the fields of chemicals, energy, and the environment. Based on the summary and perspectives, this work should contribute to the rational design of perfect metal SACs with versatile properties.

## Graphical abstract

**The marriage of single metal atoms and 2D materials** yields powerful nanoplatforms. The unique advantages, how to synthesize and characterize various 2D material‐supported metal single‐atoms and their diverse applications in chemical production, energy, and the environment are reviewed. The recent advances and research gaps of this emerging field are also summarized.

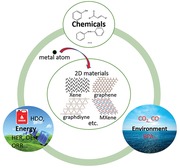

## 1 Introduction

Supported atomically dispersed metal catalysts, or metal single‐atom catalysts (SACs) have become a new frontier in field of heterogeneous catalysis, due to the breakthroughs they have achieved in the past few years.^1^ SACs refer to a series of supported metal catalysts where metals are monodispersed as separated atoms on the surface or anchored in the skeleton of a given support such as oxides,^2^ carbon materials,^3^ and metal sulfides.^4^ Supported metal catalysts, especially noble metal catalysts are most widely employed in industry for producing high value‐added chemicals (e.g., hydrogenation of nitroarenes to aromatic amines or azobenzene),^5^ liquid fuels (hydrodeoxygenation of ketones, etc),[qv: 3c] dealing with pollutants (CO, NO*_x_*),^6^ and the like. However, due to the scarce of noble metals on the earth, how to make the best of these precious resources remains a big issue for industry and laboratory research. In supported metal nanoparticle (NP) catalysts, only the atoms on the outer surface are accessible to reagents, indicating only part of the metal species participates in catalytic cycles and the portion of noble metals staying in the inner part are wasted. Quite a few investigations have focused on downsizing NPs into small clusters and finally into single‐atoms, to increase the ratio of surface metal atoms, thus making more atoms approachable. To an extreme, every single metal atom in SACs is exposed and functions as a catalytic center, maximizing the atomic efficiency of metals as well as playing a key role in dealing with the rare but powerful noble metals. It is noteworthy that the free energy of metal species increases with the decreasing sizes, so how to prevent the aggregation of these atomically dispersed metal species via metal–support interaction must be considered. In addition, each metal atom is spatially separated and directly interacts with the support via charge transfer, and thus the electronic structures of metal species are readily modulated, endowing the catalyst usually positively charged metal centers, extraordinary activities and adjustable selectivity toward ideal catalytic pathways. What is more important, the support components interacting with metal atoms sometimes are also involved in the catalytic cycle together with the metal centers, finely tuning the catalytic activity and selectivity.[qv: 2b,7] Therefore, SACs have been long regarded to bridge homogeneous and heterogeneous catalysis, as the solid supports can function as ligands coordinating and stabilizing metal atoms,^8^ as well as modulators for triggering exciting catalytic performance. In SACs, each catalytic center has identical coordination environment over a well‐defined support, offering an ideal platform for performing accurate theoretical calculations to understand how the catalytic cycle occurs.

Currently, there are two major challenges for fabricating metal SACs. First, an ideal metal loading for industrial use is usually higher than 1 wt%, but most of the obtained metal SACs suffer from aggregates at such a high loading. To solve this problem, introduction of supports with high surface area or having strong interaction with metal atoms is appreciated. Second, most of supports used for anchoring single metal atoms are nonuniform, and thus the heterogeneous nature makes metal atoms stays at various chemical environments, which makes the simplified mechanistic studies less accurate. Investigations on reaction mechanisms over metal SACs rely mainly on density functional theory calculations, so uniform supports are highly admired. Among various supports, 2D materials provide a smart choice for overcoming the above problems.

2D materials, such as graphene, transition metal dichalcogenides (TMDs), phosphorene,^9^ tellurene,^10^ and Mxene have received extensive attention in the field of optics, energy storage, sensor, electronic,^11^ and biomedical.^12^ With their unique advantages, 2D materials have been also applied to stabilize singly dispersed metal atoms different catalytic. The thin‐layer structure provides such materials with large surface area and huge number of anchoring sites for metal atoms, benefiting the practical fabrications of high‐loading SACs. 2D materials offer a much better nanoplatform than their counterpart 3D structures for strengthening metal–support interactions through chemical modifications. The simple structures of 2D materials create a uniform coordinating environment for metal atoms, making it ideal for performing studies into understanding the reaction fundamental issues through both experimental and theoretical techniques. Currently, 2D materials including MXene, graphitic carbon nitride (g‐C_3_N_4_), graphene,[qv: 3a,13] graphdiyne (GDY), TiO_2_ nanosheets[qv: 1d,2b] supported metal SACs manifest encouraging performance in thermal catalysis, eletrocatalysis, and photocatalysis. It has to be admitted that current studies on SACs@2D materials are still at the early stage and more support materials and applications need exploring.

Previous reviews or perspectives on 2D material‐supported SACs mainly focus either on limited materials or limited applications. In 2017, Bao and co‐workers summarized the research progress on three most common series of 2D supports: graphene, g‐C_3_N_4,_ and MoS_2_.^14^ Later, they enriched the discussion by comprehensively detailing the recent development of both metal and nonmetal SACs on these supports.^15^ Apart from these three main 2D materials, there are many new 2D materials emerging as intriguing candidates for stabilizing metal SACs. For example, graphdiyne is a promising 2D carbon material for stabilizing metal atoms, as the alkyne groups in the structure is more electron‐enriched than graphene. For graphene supported SACs, a usual solution is to introduce a heteroatom (O, N, S, etc.) to the structure acting as the anchoring sites, which may harm the original uniform structures, while for graphdiyne, the C≡C offers strong adsorption positions with specific sites for metal atoms. Meanwhile, Siahrostami et al. and He et al. reported on the progress on SACs catalyzed fuel cell applications and water splitting reactions.^16^ As to other general reviews discussing on SACs, the contents involving 2D supports are quite limited and many references are based on theoretical studies without experimental consolidation. A common principle for previous reviews is to divide the applications into thermal catalysis, electrocatalysis, and photocatalysis. Since different catalytic approaches sometimes serve for the same purpose, e.g., CO_2_ reduction to environmental benign chemicals. A new classification of the applications based on the reactions is required to depict a clearer picture on how a SACs@2D material contributes to industry. Thus, the present review summarizes the advances of SACs on all types of 2D supports that have been successfully synthesized in recent years, and divide the applications chemicals, environment and energy.

## 2 Unique Properties for the Combination of Metal Atoms and 2D Supports

Inspired by the promising application of 2D material‐supported SACs, tremendous trials have been conducted to combination 2D supports and metal atoms. One key issue is how to stabilize metal atoms on a support and localize them on specific anchoring sites. For metal‐free 2D materials such as graphene, heteroatoms (N, O, S, etc.) or defects are usually introduced to strengthen the bonding with the metal atoms, thus building up stable SAC systems. While for metal containing 2D supports like MoS_2_ and TiO_2_, metal atoms can take up the position of cations from the supports, staying on the skeleton of the supports via binding to the anions.

The strong interaction between metal atoms and 2D material‐based supports provides SACs with several unique properties. As mentioned before, 2D materials usually have large surface area and abundant sites for anchoring metal atoms, which are good platforms for preparing SACs with high metal loadings. As can be seen in **Table** 1, quite a few metal SACs over 2D supported achieved metal loadings higher than 0.5 wt%, which is not common for 3D supported ones. For example, 2D MoS_2_ was employed for stabilize 1.6 wt% atomically dispersed Pd species and 7.5 wt% of Pt atoms,^17^ while g‐C_3_N_4_ supported Ag SACs could achieve an incredible Ag loading of 10 wt%,^18^ which are impressive achievement of catalytic platforms for future industrial applications. In sharp contrast, the metal loadings have to be kept below 0.5 wt% or even 0.2 wt% over 3D supports such as TiN, mesoporous Al_2_O_3_ or FeO*_x_*. It cannot be denied that some 3D supports can also support high loading metal species, but not all of the metal atoms can participate in catalytic cycles. This might be attributed to the fact that metal species locate at the skeleton of the supports, which hinders the direct contact of reactants with catalytic active sites, while the metal species are generally anchored on the surface of the 2D supports.

**Table 1:** Characteristics of 2D materials supported metal SACs

Synthetic method	Catalyst	Metal loading [wt%]	Key characterization techniques	Refs.
ALD	Pt/graphene	1.52	HAADF‐STEM, XAS	36
	Pt/N‐doped graphene	>5	HAADF‐STEM, XAS	37
	Pd/graphene	0.25	HAADF‐STEM, XAS	[qv: 3a]
	Pt/N‐doped graphene	2.1	HAADF‐STEM, XAS	38
	Dy/graphene	N.A.	STM, XAS	39
	Co/g‐C_3_N_4_	1.0	HAADF‐STEM, XAS	40
	Pd/g‐C_3_N_4_	0.5	HAADF‐STEM	41
	Co/graphene	2.5	HAADF‐STEM, XAS	42
Pyrolysis	Co–N*_x_*–C	1.2	HRTEM, XPS	43
	Co–N*_x_*–C	2.7	HAADF‐STEM, XAS	44
	Cu–N*_x_*–C	8.5	HAADF‐STEM, STM, XAS	35
	Ag/g‐C_3_N_4_	10	HAADF‐STEM, solid NMR	18
	Pt/g‐C_3_N_4_	0.18	XAS	45
	Co–N_4_–C	3.6	HAADF‐STEM, XAS, Mössbauer spectra for Fe	[qv: 5b]
	Fe–N_4_–C	1.8		46
	Ni–N_4_–C	7.5		47
	M–N_4_–C (M = Fe, Co, Ni, Mn)	0.9	HAADF‐STEM	48
	Ni/g‐C_3_N_4_	2.4	HAADF‐STEM, XAS	49
	M/g‐C_3_N_4_ (M = Pd, Pt, Ir, Ag)	0.5	HAADF‐STEM	50
	Fe–N*_x_*–C	N.A.	AFM	51
	Mo_1_N_1_C_2_/N‐doped carbon	1.32	HAADF‐STEM, XAS	52
	Co/g‐C_3_N_4_	0.4	HAADF‐STEM, XAS	53
	Co–N*_x_*–C	0.25	TEM, high‐ resolution XPS	54
	M–N_4_–C (M = Co, Fe, Cu)	2.2	HAADF‐STEM, XAS	55
	Ni−N_4_–C (Topo transformation)	1.4	HAADF‐STEM, XAS	56
	Mn–N*_x_*–C	10	HAADF‐STEM, XAS	57
	M–N_4_–C (M = Fe, Ni, Pt)	4 for Fe, 9.26 for Pt	HAADF‐STEM, XAS	58
	Fe–N_4_–C	1.25	HAADF‐STEM, XAS	59
	Ni–N_4_–C	7.5	HAADF‐STEM, XAS	47, 60
	Fe/g‐C_3_N_4_	18.2	HAADF‐STEM, XAS, Mössbauer spectra	[qv: 11c]
	M–N_4_‐C_4_ (M = Fe, Co, Ni)	0.1	HAADF‐STEM, XAS	29
Wet impregnation	Pd/g‐C_3_N_4_	0.5	HAADF‐STEM, XAS	61
	Ru/Pd nanoribbon	5.9	HAADF‐STEM, XAS, SRPES	62
	Pt–S_4_–C	5	HAADF‐STEM, XAS	4
	Pt/g‐C_3_N_4_	0.16	HAADF‐STEM, XAS	63
	Pt/LDH (Zr, Sn)	0.3	HAADF‐STEM	64
	Rh/CoO (Ion exchange)	0.2	HAADF‐STEM, XAS, CO DRIFT	65
	Au/g‐C_3_N_4_	0.7	HAADF‐STEM, XAS	66
	Ni–N_4_–C (Ion adsorption)	0.8	HAADF‐STEM, XAS	67
	Fe/graphdiyne	0.63	HAADF‐STEM, XAS	40, 68
	Pt/C_3_N_4_	0.11	HAADF‐STEM, XAS, CO DRIFT	69
	Pt/Fe–N–C	2.1	HAADF‐STEM, XAS	70
	Ru/g‑C_3_N_4_	0.10	HAADF‐STEM, XAS	[qv: 3c]
Nanoparticle leaching	Ni/graphene	4–8	HAADF‐STEM, XAS	71
	Ni/graphene defects	1.24	HAADF‐STEM, XAS	10
Photochemical method	Pd/TiO_2_	1.5	HAADF‐STEM, XAS, CO DRIFT	[qv: 2b]
	Pt/N‐vacancy‐rich C_3_N_4_ (icing assisted)	2.3	HAADF‐STEM, XAS	72
	Pt/graphene	N.A.	HAADF‐STEM, XAS	25
Ball mill	Fe–N_4_–C	4.0	HAADF‐STEM, XAS, STM	13, 26
	Co–N4–C Mn–N4–C Fe–N4–C Ni–N4–C Cu–N4–C	2.6	HAADF‐STEM, XAS	27
Eletrochemical method	Ni/graphdiyne	0.28	HAADF‐STEM, XAS	73
	Fe/graphdiyne	0.68		
	Pt/Mo_2_TiC_2_T*_x_*	1.2	HAADF‐STEM, XAS	29
	Pd/graphdiyne	0.2	HAADF‐STEM, XAS	30
	M/MoS_2_ (M = Au, Pt, Pd) M/2H‐MoS_2_	>2.0	HAADF‐STEM	31
	Au/NiFe LDH	0.4	HAADF‐STEM, XAS	74
Deposition/precipitation	Ru/LDH	7.0	HAADF‐STEM, XAS	75
	Pd/g‐C_3_N_4_ (microwave)	0.5	HAADF‐STEM	76
	Co/C_3_N_4_	0.25	XAS	77
	Au/defective TiO_2_ nanosheet	0.25	HAADF‐STEM, XAS	78
	Ru/LDH	0.36	XAS	79
Physical vapor deposition	Pt/Cu_2_O film	N.A.	STM, CO DRIFT	80
	M/MgO (M = Ca, Ho, Au, Co, Fe)	N.A.	STM	81
Solvothermal synthesis	M/MoS_2_ (M = Zn, Ni, Fe, Cu, Co)	4.33	HRTEM, XRD	82
	Co/MoS_2_	1.8	HAADF‐STEM	83
Cocoon silk chemistry strategy	M–N_4_–C (M = Fe, Co, Ni)	N.A.	HAADF‐STEM, XAS	28
Plasma sputtering	Pt/graphene	N.A.	HAADF‐STEM	84

Compared to SACs where metal atoms are anchored on 3D supports, metal atoms are usually less coordinatively saturated and thus have higher chance to interact with the reactants, promoting the catalytic performance. On the other hand, 2D supports make it easier for reactants to approach catalytic centers form either sides of the support layer, which is more efficient than 3D supports in terms of mass transportation.

Finally, the well‐defined and uniform structure of 2D materials benefits the investigation of the coordination environmental of metal atoms, which further helps understand the catalytic mechanisms of important reactions through in situ techniques and theoretical studies. For 3D supports which have heterogeneous structures, many of the mechanistic studies are based on simplification and assumption, so the conclusions may not convincingly illustrate the real scenario during catalysis. 2D material‐supported SACs (SACs@2D materials) are therefore good model systems for unveiling the fundamental issues of heterogeneous catalysis.

## 3 Synthesis of SACs@2D Materials

The advantages of SACs@2D materials attract increasing attention, and tremendous effect has been devoted for developing effective synthetic approaches. Currently, 2D supports including carbon materials (graphene, nitrogen‐doped carbon, g‐C_3_N_4_, graphdiyne), metal oxide nanosheets (CoO, TiO_2_), metal oxide thin film, layered double hydroxide (LDH) and MXene have all been employed for successfully confining metal atoms through experimental techniques (Table 1). 3D material‐supported SACs can be prepared using numerous methods such as coprecipitation,[qv: 1e,19] self‐assembly,[qv: 7a,20] atom trapping,[qv: 2d,21] wet‐impregnation,^22^ and atomic layer deposition.[qv: 6a,23] Unlike 3D materials, 2D materials do not have the microporous structures for confining metal atoms, and the types of anchoring sites are not as many. Thus, the number of successful cases for 2D material‐supported SACs is still limited compared to those on 3D supports. As shown in Table 1, the most widely employed approaches are pyrolysis, ALD, wet‐chemistry strategy, and electronic deposition. Apart from these common methods, there are also different effect ways for SACs preparation. For example, physical or chemical vapor depositions have been broadly applied for preparing model metal SACs on ultrathin metal or metal oxide films, which helps to understand fundamentals of catalysis accurately.^24^ However, SACs prepared this method is not suitable for industrial applications due to the expensive costs compared to methods discussed above and narrow range of candidates. In this part, several interesting facile methods for preparing metal SACs over 2D supported will be introduced.

Considering that the common synthetic methods of SACs have been discussed in depth in previous reviews, this review focuses on some novel methods including photochemical synthesis, ball milling method, cocoon silk chemistry strategy and electrochemical process.

### 3.1 Photochemical Synthesis

**Figure 1:**
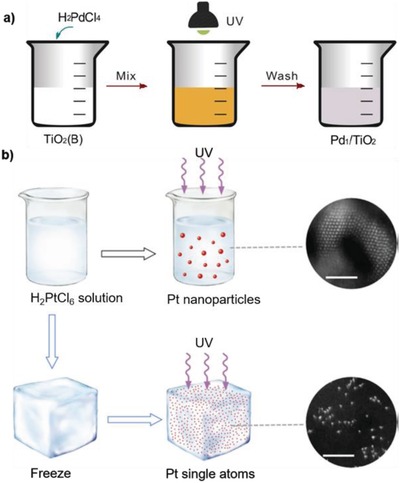
a) Schematic synthesis of Pd_1_/TiO_2_. Reproduced with permission.[qv: 2b] Copyright 2016, AAAS. b) Schematic synthesis of iced‐photochemical process. Reproduced with permission.^25^ Copyright 2017, Nature Publishing Group.

A facile method has been applied for SACs synthesis is photochemical process. Zheng's group first reported this method for fabricating Pd SACs over 2D TiO_2_ nanosheets, and the process is described in **Figure** 1.[qv: 2b] 1.5 wt% Pd are atomically dispersed onto ethylene glycolate stabilized ultrathin TiO_2_ nanosheets via this method. During the preparation process, ethylene glycolate radicals are formed on the surface of TiO_2_ after exposure to ultraviolet light. It is proposed in this study that the formed radicals promote the formation of PdCl_1_/TiO_2_, which is an intermediate that can be readily converted to the Pd_1_/TiO_2_ SAC. The obtained Pd SAC shows exiting catalytic performance in the hydrogenation of C=C and C=O groups. Another example using photochemical approach is to freeze the aqueous solution of H_2_PtCl_6_, followed by irritating the solution at low temperature with UV light. Amazingly, a Pt single atom solution was obtained before loading onto a graphene support or other supports.^25^ Considering that phtotochemical method requires photochemical process, a major issue that should be concerned is whether this method can be extended to other metals or supports. Further, this method is performed under a mild condition, and thus whether the SACs can be applied to catalysis at high temperatures is to be proved.

### 3.2 Ball Milling Method

**Figure 2:**
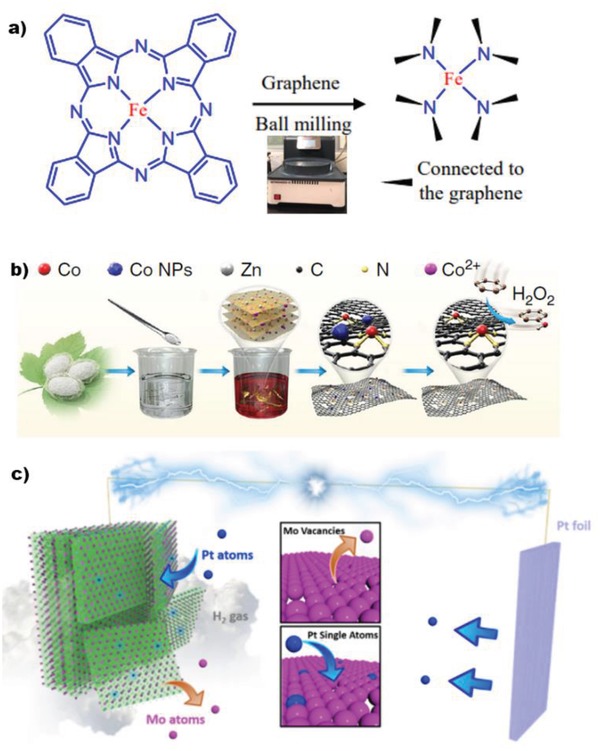
a) Ball milling method for synthesizing Fe SAC on graphene. Reproduced with permission.^13^ Copyright 2015, AAAS; b) Schematic illustration of the cocoon silk chemistry strategy for preparing Co SACs on N‐doped C nanosheet. Reproduced with permission.^28^ Copyright 2018, Nature Publishing Group; c) Schematic illustration of electrochemical exfoliation process of MXene with immobilized Pt single atoms. Reproduced with permission.^29^ Copyright 2018, Nature Publishing Group.

Ball milling using steel balls to mill the Fe precursor with graphene, especially under high energy is proved to achieve highly catalytic active Fe SACs on graphene, because high energy ball milling created defects for anchoring metal atoms during the milling process (**Figure** 2a). Bao and his co‐workers applied this technique to synthesize high loading Fe SACs, which showed excellent activity for benzene oxidation, methane conversion, and oxygen reduction reactions (ORRs).^13^, ^26^ Further, they extended the metal scopes to various transitional metals (Co, Mn, Cu, etc.), all of which exhibited potential for dye‐sensitive solar cells.^27^

### 3.3 Cocoon Silk Chemistry Strategy

This strategy is inspired by the chemical composition of silk fibroin, which is enriched in amino groups and geometric sheet structure, which is important for the construction of 2D materials with rich N sites for anchoring metal atoms. It involves four steps (Figure 2b): 1) extraction of degummed silk fibroin; 2) regeneration of layered‐structure silk fibroin in the aqueous solution containing metal salts; 3) pyrolysis to yield 2D materials and convert amino group to N‐metal bonds; and 4) removal of metal NPs with acid.^28^ With this smart approach, Li and co‐workers achieved highly efficient transition metal SACs on N doped carbon nanosheet for benzene oxidation to phenol through C‐H activation at room temperature.

### 3.4 Electrochemical Process

As shown in Figure 2c, it is easy to correlate SACs obtained via such an electrochemical process with electrocatalytic applications, because such SACs are on the electrode. This approach has been utilized for preparing MXene,^29^ graphdiyne,^30^ and MoS_2_ 31 supported metal SACs, which also exhibited broad range of candidates. Taking Pt/MXene as an example, Mo_2_TiC_2_T*_x_*MXene was obtained after etching Mo_2_TiAlC_2_ with HF. A system equipped with three electrodes was used Pt foil at the counter electrode and carbon supported MXene function as at working electrode. MXene that is originally packed underwent exfoliation to nanosheets in this process. Simultaneously, Mo atoms left the MXenes and Pt atoms that had been detached from the Pt foil moved to the working electrode, filling in the vacancy created by the missing Mo. Different types of metal SACs have been prepared, but they are mainly devoted to electrocatalysis such as hydrogen evolution reaction (HER), which requires broadening the application ranges.

### 3.5 The Remaining Problems for Constructing SACs@2D Materials

Frankly speaking, although various methods for synthesizing metal SAC@2D materials have been developed, they are derived from the approaches for SAC@3D supports such as pyrolysis and coprecipitation. The most widely utilized strategy for stabilizing metal atoms on 2D supports is pyrolysis, which helps anchor high loading metal species onto graphitic carbon materials. However, pyrolysis generally results nonuniform structures, and the heteroatoms and carbon atoms probably do not follow a regular arrangement. What is more, high temperature treatment might lead to agglomeration of metal atoms and small portion of aggregates are beyond the detection limit of most characterization techniques in current studies. This makes such SAC@2D materials also suffer from heterogeneous issues commonly faced in 3D supports, and makes it challenging to fundamentally understand the working principle of SACs. Novel approaches including icing‐assisted photocatalytic reduction and electrochemical methods are adopted for fabricating uniform SACs, but they are limited to specific systems and the applications are quite narrow. Developing smart methods for well‐defined metal SAC@2D materials with general applications remains a big challenge.

## 4 Characterization Techniques of Metal SACs@2D Materials

SACs only contain isolated metal atoms that perform as separate catalytically active centers, so how to identify the existence of these atomically dispersed metal species and determine their distributions as well as the coordination environments are critical for investigating metal SACs. In recent studies, sophisticated characterization tools used for that purpose include electron microscopy,[qv: 6a,20,32] especially aberration‐corrected high‐angle annular dark‐field scanning transmission electron microscopy (HAADF‐STEM) and scanning tunneling microscopy (STM), as well as spectroscopic techniques,[qv: 1b,6b,7b,20,32b,33] including X‐ray absorption spectroscopy (XAS) including extended X‐ray absorption fine structure (EXAFS), X‐ray absorption near edge structure spectroscopies (XANES), infrared (IR) spectroscopy, H_2_‐O_2_ titration and solid nuclear magnetic resonance (NMR) spectroscopy. Among these techniques, HAADF‐STEM, STM, XAS, probe molecule‐IR are generally adopted as the direct tools for identifying the single‐atom identity. Other techniques, such as transmission electron microscopy (TEM) with EDX, H_2_ titration, X‐ray photoelectron spectroscopy, X‐ray diffraction (XRD) cannot provide solid evidence for singly dispersed metal atoms, by can indirectly indicate whether the materials are NPs. To convincingly prove the formation of metal SACs, at least one of the direct characterization techniques is required. The applications of the techniques are introduced below and the challenges will be discussed, and techniques exclusively for characterizing the 2D supports are not discussed here.

### 4.1 Direct Approach

**Figure 3:**
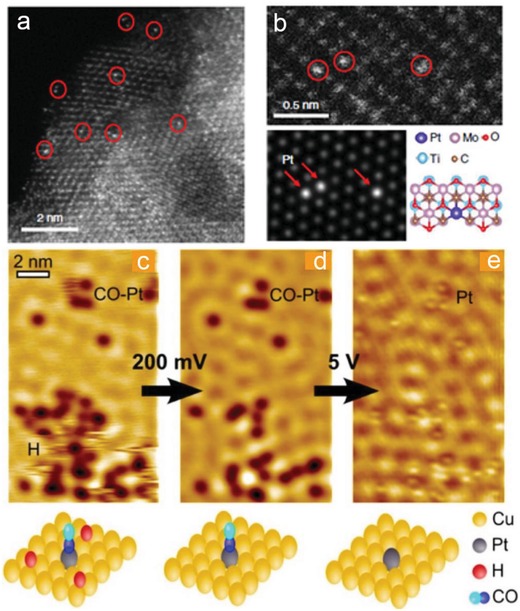
a,b) An HAADF‐STEM image of Mo_2_TiC_2_T*_x_*–Pt_SA_. Reproduced with permission.^29^ Copyright 2018, Nature Publishing Group. c–e) STM images showing the coadsorption of H and CO on a Pt–Cu (111) SAA surface and STM tip‐induced adsorbate removal to reveal the binding sites beneath. Reproduced with permission.[qv: 32c] Copyright 2013, American Chemical Society.

Two microscopy techniques, HAADF‐STEM and STM,^34^ make it possible to directly image the individual atoms on the supports and have been applied to characterize metal SACs. As long as the central metal atoms are significantly heavier than atoms in supports, we will be able to detect the location and distribution of the metal atoms or NPs via HAADF‐STEM. This method is extremely important for confirming the existence of only single metal atoms without the presence of any metal NCs or NPs, identifying the location of the single metal atoms with respect to the surface structure of the support, as well as determining the spatial distribution of the single metal atoms. As shown in **Figure** 3a,b, Li's group identified Pt single atoms and NCs over Mo_2_TiC_2_T_x_–Pt_SA_nanosheet, which helps understand the coordination and distribution of Pt atoms.^29^

**Figure 4:**
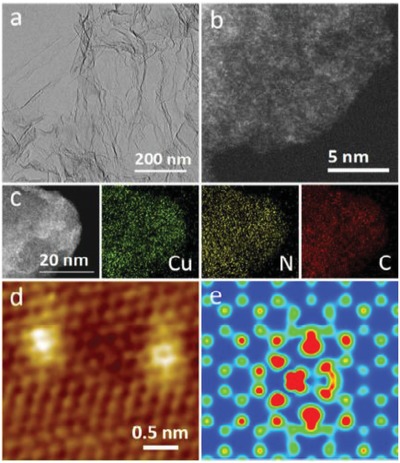
Morphology and structure of Cu‐N–C‐60. a) TEM image. b) HAADF‐STEM image. c) HAADF‐STEM image and the corresponding element mappings for Cu, N, and C atoms d) STM, and e) STM simulation images. Reproduced with permission.^35^ Copyright 2016, Royal Society of Chemistry.

STM, another electron microscopy tool, can provide both the atomic and electronic structure information of single metal atoms only when the underlying supports are conductive. For instance, Maria and co‐workers employed STM images to confirm the single‐atom identity of their Pt/Cu SAC and investigate the nature of H and CO adsorption sites, demonstrating that H adatoms are capable of diffusing onto Cu sites and away from the Pt dissociation site.[qv: 32c] Bao and co‐workers checked the Cu SACs on N‐doped graphene with a combination of HAADF‐STEM and STM (**Figure** 4), which confirmed the existence of atomically dispersed Cu species and suggested the Cu–N complex is in the graphene lattice, which provided convincing information for deciphering the SAC structures.^35^

**Figure 5:**
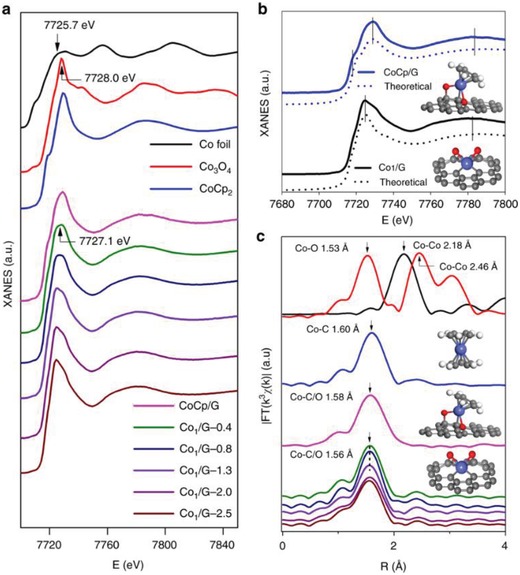
Co K‐edge XAFS and EXAFS spectra of CoCp/G, Co1/G SACs. a) Co K‐edge XANES spectra. b) The experimental XANES curves and calculated XANES data of optimized DFT‐modeled structures of Co1/G and CoCp/G. c) Fourier transform EXAFS of these samples with the corresponding structures (insets). The balls in gray, white, red, and blue represent carbon, hydrogen, oxygen, and cobalt, respectively. Reproduced with permission.^42^ Copyright 2018, Nature Publishing Group.

**Figure 6:**
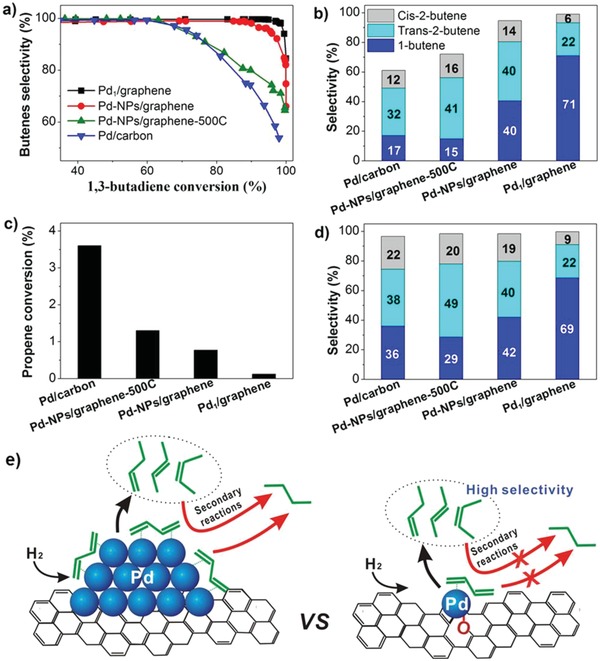
Catalytic performances of Pd1/graphene, Pd–NPs/graphene, Pd–NPs/graphene‐500C, and Pd/carbon samples in selective hydrogenation of 1,3‐butadiene. a) Butenes selectivity as a function of conversion by changing the reaction temperatures; b) the distribution of butenes at 95% conversion. c) Propene conversion and d) the distribution of butenes at 98% 1,3‐butadiene conversion in hydrogenation of 1,3‐butadiene in the presence of propene. e) A schematic illustration of improvement of butenes selectivity on single‐atom Pd1/graphene catalyst. Reproduced with permission.[qv: 3a] Copyright 2015, American Chemical Society.

We have to admit that the microscopic tools cannot provide conclusive information on whether no metal NPs are formed because those techniques cannot examine every area of the sample. To gain more detailed information of the structural features of the single atoms, XAS, especially EXAFS, studies have been performed to convincingly illustrate the contribution of different coordination to the metal atoms.[qv: 2a,85] EXAFS spectra arise because of the interference effects between the outgoing wave and the backscattered wave produced by the surrounding atoms around the central atom, which provides the average information of the whole sample.^86^ From the analysis of EXAFS data, information about the local coordination number, interatomic distances, structural disorder, and kind of neighboring atoms at a provided bond length can be obtained (**Table** 2). For SACs, the metal–metal bonding should be absent in their EXAFS spectra. Thus, if the EXAFS results do not contain any signal on the metal−metal shell, by inference, it can be concluded that only isolated metal atoms are present in the catalyst. On the other hand, XANES tells the electronic and geometric coordination structures of metal species based on the white‐line intensity as well as the finger‐print peaks features. A common analysis procedure using XAS is to confirm the single metal atom identity with EXAFS and quantitative coordinating results via result fitting, followed by curve‐fitting of XANES based on possible coordination environment to understand the geometric configurations. As shown in **Figure** 5c, no Co–Co bonding was formed for all the Cobalt catalysts, suggesting the single‐atom identity throughout the whole sample. Analysis of the white‐line intensity values in Figure 5a showed that the chemical valence of Co in the Co SACs are lower than +2, but Co species are positively charged, suggesting the electronic interaction between Co and the support. Further curve‐fitting in **Figure** 6b provided the optimized structure of this Co SAC.

**Table 2:** The comparison of EXAFS Fitting results and the optimized model of CoCp/G and Co_1_/G samples

EXAFS fitting						
Sample	Shell		C.N.		*R* [Å]	σ^2^ × 10 [Å^2^]
CoCp_2_	Co–C		10.0		2.04	2.6
CoCp/G	Co–O		2.7		1.95	0.9
	Co–C		4.8		2.10	1.7
Co_1_/G	Co–O		2.7		1.04	1.4
	Co–C		4.2		2.09	1.4
DFT						
				*R*		*N*
CoCp/G		Co–O		2.00		2
		Co–C		2.05		6
Co_1_/G		Co–O		1.90		2
		Co–C		2.03		4

C.N., coordination number; R, bond length; σ^2^, the Debye–Waller factor. Errors in the fitting parameters are N ± 20%, R ± 0.02 Å, σ^2^ ± 20%. Reproduced with permission.^42^ Copyright 2018, Nature Publishing Group.

**Figure 7:**
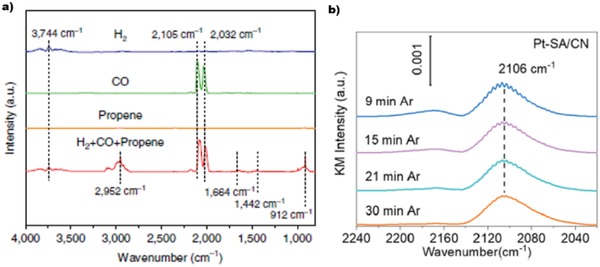
a) In situ DRIFT spectra of 0.2%Rh/CoO after the treatment of the sample with different gas at 100 °C. The peaks at 2105 and 2032 cm^−1^ correspond to the symmetric and antisymmetric stretching vibrations of CO in Rh(CO)_2_, respectively. Reproduced with permission.^65^ Copyright 2016, Nature Publishing Group. b) DRIFTS of CO adsorbed on Pt‐SA/CN after being purged with Ar gas for different time. Reproduced with permission.^69^ Copyright 2018, American Chemical Society.

Probe molecules infrared (IR) spectroscopy is another powerful tool to evaluate the existence of metal atoms and, to a certain degree, to quantify the concentration of single‐atom species in a supported metal catalyst.^87^ This method studies the interaction between the probe molecules and the metal surface to obtain information on the nature of the catalyst. By monitoring the changes in vibrational frequency and intensity of the probe modes one can, with proper calibration, deduce the properties of the active centers. Recently, as an in situ technique, diffuse reflectance infrared Fourier transform spectroscopy with CO as the probe (CO‐DRIFTS) containing known site‐specific extinction coefficients has been employed to investigate the percentage of isolated atoms in supported metal catalysts that consist of both single atoms and NPs quantitatively.[qv: 7b,33c] However, quite a few 2D materials such as graphene and graphdiyne are dark in color, making it difficult to get high‐quality IR signals over these samples, thus hindering its application for 2D material‐supported SACs. CO‐DRIFT has also been employed to confirm the metal existing form in SACs on 2D CoO, TiO_2,_ and C_3_N_4_. For example in **Figure** 7a, Zeng's group observed a pair of peaks corresponding to the symmetric and antisymmetric vibration stretching of CO in Rh(CO)_2_^3+^, which confirmed that Rh exists as single atoms.^65^ For Rh and Ir, their cations prefer to absorb two CO molecules, while their metallic species only absorb only one CO, which only has one IR absorption peak. Thus, CO‐DRIFT is very sensitive for differentiating SACs and NPs. In terms of metals whose cations only absorb one CO, the CO absorption peaks have two characteristics: a) the peak position undergoes blueshift from corresponding metal NPs; b) the peak on isolated metal sites are symmetric narrow peaks. As shown in Figure 7b, the peak position of Pt single atoms on g‐C_3_N_4_ is 2106 cm^−1^, which is 20 cm^−1^ than CO on the corresponding Pt NPs. Another disadvantage of CO‐DRIFT is that it is not applicable to metals, which have weak interaction with CO.

### 4.2 Indirect Characterization Techniques

#### 4.2.1 Gas Chemisorption

**Figure 8:**
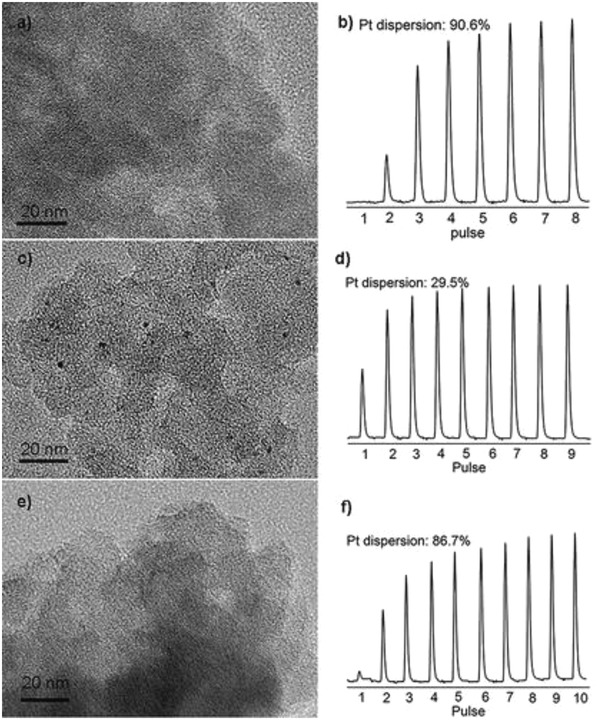
a,e) TEM images and b,f) corresponding H_2_ pulse titration profiles of two carbon materials supported Pt SACs. c,d) TEM image and corresponding H_2_ pulse titration profile of one carbon materials supported Pt SACs. The titration ends as the peak intensities reach a constant value. Reproduced with permission.[qv: 88b] Copyright 2019, American Chemical Society.

Surface atom dispersion of a metal catalyst is an important term for studying heterogeneous catalysis, as it determines the turnover frequency (TOF). Gas chemisorption such as CO chemisorption or H_2_–O_2_ chemisorption can be employed to determine the number of the metal atoms on the surface. For metal NPs, the surface atom percentage should be lower than 100% due to the atoms hidden by the surface ones, while the dispersion should be 100% for SACs or small clusters with several metal atoms in principle.^20^, ^88^ **Figure** 8 provides an example of carbon‐supported single atom and NPs at the same loading, which showed obviously that the dispersion is much higher than that of NPs.[qv: 5c] This provides a facile way to check whether large metal NPs were formed in a given catalyst. However, in real cases, the measured values may not necessarily provide accurate information for the dispersion, as the gas chemisorption is quite complex (e.g., Rh^+^ absorbs two CO molecules while Rh^0^ absorbs one).

#### 4.2.2 TEM and XRD

The detection limit for normal TEM is ≈1 nm and that of XRD is about 3 nm. When characterizing metal SACs, what researchers expect is to observe no metal NPs using these two techniques. The combination of TEM and EDX is usually employed to exclude the existence of metal NPs in the given area. No large NPs were detected with the high‐resolution TEM, but this might be due to the area does not have the desired metal element. Therefore, EDX mapping of the material is crucial for proving the existence of desired metals and the distribution of different elements. The conclusion obtained from XRD result is even weaker, due to the higher detection limit and demands for higher metal loadings.

#### 4.2.3 XPS

XPS is employed for showing the chemical valence of surface elements, as well as depicting the elemental composition of the surface, which helps to identify whether the metal atoms exist in the near surface of the material. This technique also suffers from low signal to noise ratio at low metal loadings, which can be solved over high‐loading 2D material‐supported SACs. Considering that the metal species are usually positive charged due to the interaction with supports and that the anchoring sites of 2D supports are supposed to be uniform, XPS helps to identify whether the metal elements have a unified positive charge with a singlet peak in XPS results. However, this technique has difficulty in differentiating metal oxide NPs with single metal atoms. Besides, due to the heterogeneity of supported SACs in reality, nonuniform electronic structures may not necessarily be ascribed to the formation of metal NPs.

A comparison between different techniques for judging whether a given metal catalyst is SAC or not is listed in **Table** 3. To conclude, every technique has its pros and cons, so combining different techniques are of high significance for convincing evaluating a catalyst. Apart from the ex situ modes, characterization techniques operating in situ are more useful for providing an accurate understanding of the SACs.

**Table 3:** A summary of the major characterizations techniques for proving the existence of single‐atom identity

Techniques	Advantages	Disadvantages
AC‐STEM	Straightforward	Statistically limited, only providing 2D instead of 3D projection information
XAF	Providing information of the whole sample, electronic state, and coordination environment	Average information, not conclusive for every atomic center
CO‐DRIFT	Site‐specific, information on electronic state	Charge‐dependent, SAC, and small NP might not be easily differentiated, not applicable for metals with bad adsorption for CO and dark‐colored supports
STM	Straightforward	Statistically limited, not applicable for samples with poor conductivity
Titration	General approach, providing information of exposed metal atoms	Indirect and inconclusive

## 5 Catalytic Applications of Metal SACs over 2D Materials

In the past few years, versatile applications of 2D material‐supported SACs have been explored through rational material design, which convincingly demonstrated that the single metal atoms instead of metal NPs are the key active centers. Benefiting from the strong interaction between isolated metal atoms and anchoring sites on 2D supports, these SACs in many catalytic reactions excel their corresponding NPs in both catalytic performance and stability. Unlike SACs@3D supports, the combination of metal single atoms and 2D materials contributes greatly to the fundamental understanding of heterogeneous catalysis, opening the window to broad applications of these frontier catalysts. In this section, the applications of 2D material‐supported SACs in chemistry, energy, and environment will be introduced and discussed.

### 5.1 Application of SAC@2D Materials for Value‐Added Chemical Production

Heterogeneous catalysis plays a significant role in producing value‐added fine chemicals and chemical purifications from raw materials with low values or wastes. Due to the special coordination structure with solid supports, supported metal single atoms usually catalyze the reaction of a certain functional group to a uniform extend, which have already achieved good results in producing a variety of high‐value chemicals.

#### 5.1.1 Production of Chemicals from Nitroarenes

**Scheme 1:**
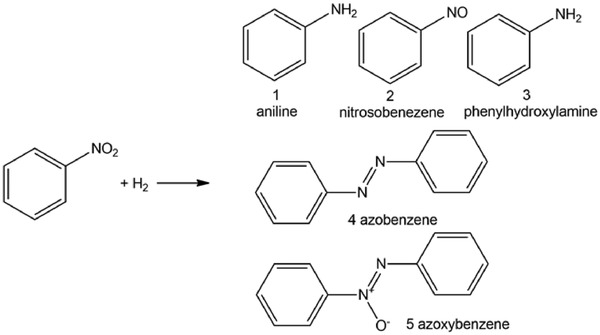
Hydrogenation of nitrobenzene to five possible products.

Aromatic amines, azo and azoxy compounds are important chemicals and processing intermediates in the field of organic synthesis, pharmaceuticals, and dyes.^89^ Selective hydrogenation of nitroarenes that are inexpensive to one desired product has been widely employed to produce these chemicals. Taking the hydrogenation of nitrobenzene of as an example, different products distributions were obtained on metal SACs and NPs using the same support (**Scheme** 1). For NPs, the metal sites at the surface are not uniform, and different sites might work through different mechanisms, yielding different products at the same time. For metal SACs, selective hydrogenation of nitrobenzene toward aniline, azobenenze, or azoxybenezene has been achieved with appropriate catalysts due to the identical chemical environmental of each metal atoms. The products distribution over 3D supported SACs vary.

At the optimal condition for nitrobenzene hydrogenation, aniline production on both noble metal and non‐noble metal SACs were achieved with 100% selectivity, as shown in **Table** 4. Although the TOF value over Pt SAC (774 h^−1^) is slightly higher than that over Co SAC (589 h^−1^), the cheaper cobalt is more promising for practical application. Aniline is the product after complete hydrogenation of nitrobenzene, thus 100% selectivity at full conversion in principle is not difficult. In the work conducted by Zhang et al., the 100% selectivity toward aniline was obtained at only 30% conversion over a Pt SAC, which demonstrated the superior properties of metal SACs over 2D supports.[qv: 5c] Furthermore, Pérez‐Ramírez et al. found that a single Pd atom on graphitic carbon nitride was also selective toward the aniline in a continuous flow system. Compared to the result over Pt SAC@3D FeO*_x_* support, which also achieved high selectivity toward aniline, Pt SAC@2D carbon support loads 10 times more Pt atoms with comparable TOF values.[qv: 5a]

**Table 4:** List of metal SACs on 2D supports for hydrogenation of nitrobenzene

No	Catalyst	*T* [°C]	S/C ratio	TOF [h^−1^]	S to 1 [%]	S to 4 [%]	S to 5 [%]	Refs.
1	Pt‐PMA/C	25	2000	774	100	0	0	[qv: 5c]
2	Pt(acac)_2_–PMA/C	25	2000	0	–	–	–	[qv: 5c]
3	Co–N_4_/C	80	143	95.3	99	0	0	[qv: 5b]
4	Co_1_/G	25	71.4	1188	–	–	65	42
5	Co–N*_x_*/C‐600	110	409	36.8	>99	0	0	54
6	Co–N*_x_*/C‐800	110	589	589	>99	0	0	54
7	Co–N*_x_*/C‐900	110	818	579	>99	0	0	54
8	0.2Pt/m‐Al_2_O_3_ (NP)	25	2000	2000	100	0	0	20
9	0.2Pt/p‐Al_2_O_3_ (NP)	25	2000	1450	62	2.7	34.2	20
10	Pt/AC (NP)	25	2000	917	50.9	2.7	1.8	[qv: 5c]

**Figure 9:**
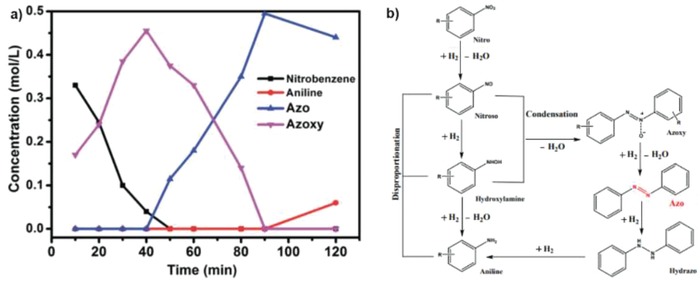
a) Substrate and product concentration profiles as a function of the reaction time; b) proposed pathways for the reduction of nitroarenes on Co‐N_4_‐C. Reproduced with permission.[qv: 5b] Copyright 2016, Royal Society of Chemistry.

On the other hand, Liu et al. and Yan et al. performed managed to obtain the two coupling compounds with cobalt SACs.[qv: 5b] Considering that azo and azoxy are the intermediate products during nitrobenzene hydrogenation, metals that are less active are required to inhibit the complete hydrogenation process. As shown in Table 3, a suitable solvent is highly important for the exclusively production of azobenezene, with *tert*‐butanol as the desired candidate for the single Co atom coordinating with the N_4_ site on graphitic carbon. To check the reaction mechanism, reaction kinetic studies with optimized parameters. Based on the kinetic results in **Figure** 9a, azoxybenzene was believed to be the intermediate before the production of azobenzene and aniline, which matched well with a condensation reaction pathway in Figure 9b. This group further extended the substrate scope to different nitroarenes and found that their catalyst had exceptional selectivity toward azo compounds regardless of the electron‐rich or electron‐defective groups on the aromatic ring. In entry 4 of Table 3, it is interesting to see that NaBH_4_ was adopted as the hydrogen source, which might be due to the low reactivity toward the azoxy product. Besides, a mixture solvent of THF and water increased the complexity of the catalytic system, hindering its application for industrial application.

It is worth noting that 3D supports supported metal SACs might yields a broad product distribution, which is the case for 0.2Pt/p‐Al_2_O_3_. Entry 8–9 in Table 3 showed that the two Al_2_O_3_‐supported Pt SACs have different products, and the only difference for the two supports are the coordinative environment. m‐Al_2_O_3_ is rich in pentahedral Al^3+^ centers that have strong affinity toward to Pt atoms. The relatively uniform structures of 2D supports make SACs@2D supports good platforms for producing high‐purity chemicals, which surpasses 3D supports in some cases. However, due to the unique metal–support interaction between metal species and 3D materials, the TOF and stability of SACs@3D supports are usually high.

#### 5.1.2 Production of Alkenes from the Dienes and Alkynes

Alkenes belong to key starting materials and intermediates for synthesizing building blocks of various fragrance, plastic polymers, and vitamins. Selective hydrogenation of dienes and alkynes to olefins is a common practice for hydrorefining process of alkenes, which has attracted tremendous interest from industry.

It has been reported that butadiene metal catalysts for polymerization of alkenes could be poisoned by butadiene impurities even at a concentration of 10 ppm, which makes selective hydrogenation of butadienes to butenes without further hydrogenation of the C=C bonds to alkanes in industrial processing of the alkene streams. What makes this reaction more intriguing is that propene feedstocks which contain butadiene impurities contributed to polypropylene production with an amount of more than 40 million tons.^90^ Pd and Pt catalysts are favorable catalysts for this reaction, but usually suffer from low stability in presence of hydrogen gas and decreasing selectivity at high conversions. A common strategy to inhibit the second step hydrogenation is to form bimetallic alloys, which would increase the catalyst costs and environmental problems. To deal with this issue, Lu and co‐workers synthesized a highly efficient Pd SAC on graphene and g‐C_3_N_4_via ALD method, and applied it to catalyze the hydrogenation of 1, 3‐butadine in presence of excess propene.[qv: 3a,91]

**Figure 10:**
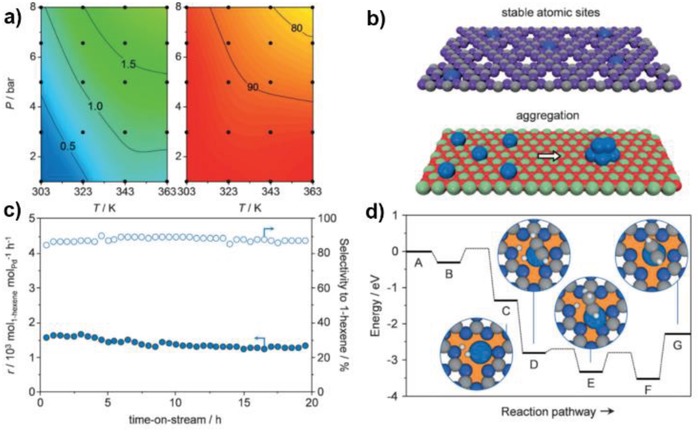
Catalytic performance in hydrogenation of 1‐hexyne over Pd_1_/g‐C_3_N_4_ SAC. a) Reaction rates, selectivity to 1‐hexene. b) Representation showing isolated Pd species on carbon nitride (top) and alumina (bottom). c) stability over the course of 20 h at 343 K and 5 bar during the hydrogenation of 1‐hexyne over [Pd]mpg‐C_3_N_4_. d) Energy profile for the hydrogenation of acetylene over a single Pd atom. Reproduced with permission.^61^ Copyright 2015, Wiley‐VCH.

**Figure 11:**
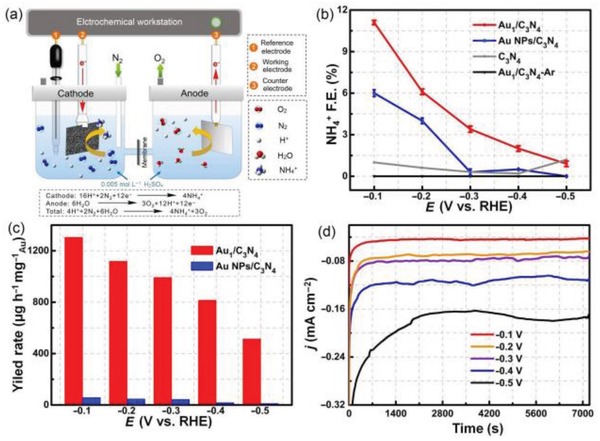
Performance of N_2_ electroreduction. a) Schematic electrolyzer for N_2_ electroreduction test. b) NH_4_^+^ formation Faradaic efficiencies for Au_1_/C_3_N_4_, Au NPs/C_3_N_4_, pure C_3_N_4_ and Au_1_/C_3_N_4_ with Ar feed instead of N_2_. c) NH_4_^+^ yield rates normalized by Au mass. d) Catalytic durability test for Au_1_/C_3_N_4_ at different potentials. Reproduced with permission.[qv: 99a] Copyright 2018, Elsevier.

As described in **Figure** 10, the Pd SAC achieved almost 100% selectivity toward butenes even at 80% conversion of butadine. Meanwhile, the hydrogenation of propene was much less prominent compared to the other Pd NPs. The authors ascribe to the difference adsorption modes of 1,3‐butadine on isolated Pd atoms and Pd assembly. As shown in **Figure** 11e,d, di‐π‐adsorption of butadiene on Pd NPs promoted the further hydrogenation of butenes to butanes, while Pd single atoms only absorb butadiene through mono‐π‐adsorption mode with increased steric hindrance, inhibiting the hydrogenation of butenes. They also conducted kinetic studies (reaction order measurement, H‐D exchange, etc) and DFT calculations to confirm the rate‐limiting step is highly influenced by the adsorption mode of substrate. Based on the comparison study between Pd SAC on graphene and g‐C_3_N_4_, the acidity and porous structures of supports as well as electronic states of Pd have all been demonstrated to effect this selective hydrogenation reaction, which lays a good theoretic foundation for designing diene‐to‐alkene catalysts.

For the hydrogenation of alkynes and 1,3‐butadine over SACs@2D supports, it seems the supports were not directly involved in the catalytic cycles based on the reported literatures. In current studies, mechanistic studies on the H_2_ splitting and surface reactions over metal atoms are in lack. Traditionally, H_2_ are split into two H atoms and stay on two neighboring metal atoms, while on SACs it is difficult to identify where the two H atoms are. Zhang et al. examined the H_2_ splitting process over polyoxometalates modified carbon materials through experimental and theoretical calculations, and the conclusion suggests H_2_ undergoes a homolytic dissociation process and H atoms do not migrate the supports.[qv: 88b] In sharp contrast, Zheng and co‐workers found that H_2_ underwent heterolytic dissociation with H^δ+^ migrates to the O species of TiO_2_ nanosheets and H^δ−^ on singly dispersed Pd atoms.[qv: 2b] Although H_2_ dissociation requires the involvement of supports, the rate‐limiting steps for various types of hydrogenation reactions lies in the elemental step where one absorbed H atom attacks the absorbed intermediate on the same metal atoms, which does not differentiate dimensions of supports. Generally, the major role of 2D supports in hydrogenation reactions are to stabilize metal atoms and endow the metal species unique electronic states, which is similar to 3D supports. The neighboring atoms on the supports do not participate in the reaction pathways. This may explain the SACs@3D supports have similar performance to that over 2D supports when the surface areas are comparable. The advantages in surface area and smaller coordination number highlight the potential of SACs@2D supports.

Various alkynes were selectively converted to alkenes over 2D material‐supported metal SACs. For this reaction over metal NPs, the surface atoms are usually modified to alter the electronic state and coordination environment to achieve high selectivity, such as introducing a second metal to form alloys and downsizing metal particles. It is generally accepted that lower coordination number among the catalytically active metal atoms favors half hydrogenation to alkenes. The catalytic performance of 2D carbon material‐supported Ag, Pd, and Pt SACs for different alkynes has been investigated, all of which come the same conclusion that SACs have higher tendency to produce valuable alkenes from alkynes. Single Pd atoms on g‐C_3_N_4_ were reported with great efficacy after exemplifying with acetylene and 1‐hexyne hydrogenation. Compared to the classic Pd hydrogenation catalysts, Pd SAC proved to have the highest TOF (1.41 × 10^3^ h^−1^) and selectivity toward 1‐hexene (90%) when performing at the same condition.^61^ Compared with Pd SACs on 3D Al_2_O_3_ support which easily form aggregates, Pd atoms dispersed on g‐C_3_N_4_ are intrinsically more stable and sintering‐resistant due to the strong bonding interaction between Pd and the N atoms. As shown in Figure 10b, no obvious losses of high production rates and selectivity toward the desired alkene product were observed even after a long time run for 20 h, which is promising for scalable applications.^61^ To explain the outstanding performance of Pd SAC, theoretical calculations of the reaction pathway were conducted based on acetylene. As depicted in Figure 10d, the heterolytic dissociation of H_2_ leaves one H atom on one Pd atom and the other on bonded N atom, which alters the aromatic of the support. This in turn tunes the adsorption energy of alkene on Pd atoms and inhibits the over‐hydrogenation, which accounts for the high selectivity.^50^ More interestingly, g‐C_3_N_4_ supported Ag single atoms with an Ag loading of 10 wt% shared a similar TOF with commercial Lindlar catalyst. Although the Ag SAC is less active than Pd SAC due to more efficient H_2_ splitting over Pd, the much higher weight loading and cheaper price is impressive.^92^ With ALD approach, Pd SACs on graphene oxide and graphitic carbon nitride were obtained showing slightly different performance in acetylene hydrogenation. Pd/graphene achieved higher conversion (80% vs 50% at 60 °C) at the same condition, but the selectivity to ethene is worse at the same conversion (80% vs 98% at 60% conversion). These phenomena were ascribed to different valences of Pd in two catalysts. Pd existed predominantly as Pd^4+^ when loaded onto carbon nitride due to the strong charge transfer from Pd to N, while the Pd species have a valence of +2 on graphene oxide. Pd atoms with lower valence tend to interact strongly with alkenes via as the d orbitals electrons of Pd can move to the empty π* antibonding molecular orbitals, which yield superior activity.[qv: 91a] Further, another important chemical, 2‐methyl‐3‐buten‐2‐ol was produced from 2‐methyl‐3‐butyn‐2‐ol with high purity over metal SACs.

To sum up, metal SACs@2D materials are highly efficient and stable for selectively producing alkenes from butadines and alkynes, compared to both conventional NP catalysts and Pt SACs@3D supports. High metal loading up to 10 wt% has been achieved for such catalysts, which offers an alternative platform for large scale application in industry. A limitation for current studies is that the reactions explored are mainly gas phase reaction, and liquid phase reactions for producing aromatic alkenes are rarely investigated on metal SACs@2D materials, which needs further exploration as solvents involved might inhibit the catalytic performance or induce metal leaching.

#### 5.1.3 Aromatic Compounds from C–C Coupling Reaction

Suzuki reaction, Ullmann reaction and Sonograshira reaction are C–C coupling reactions for connecting different substituted aromatic rings, which are generally catalyzed with homogeneous catalysts, especially Pd organometallic complex. Supported Pd NPs are also found effective for these coupling reactions. For homogeneous coupling reactions, the separation of products with the metal complex is difficult, while mechanistic studies over metal NPs are too complicated to draw a conclusion. Therefore, supported SACs, bridging homogeneous and heterogeneous catalysis, were employed for ideal platform to dig into the coupling reaction. Apart from 3D TiO_2_,^93^ 2D materials including g‐C_3_N_4_ and N‐doped graphitic carbon supported metal SACs also proved excellent activity for catalyzing Suzuki reaction and oxidative coupling reaction.[qv: 3b,94]

**Figure 12:**
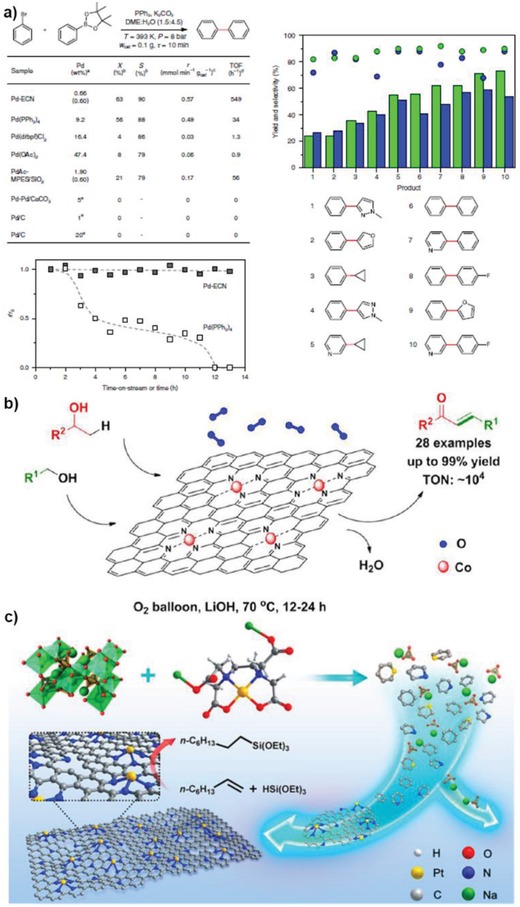
a) Evaluation of the Suzuki coupling performance of Pd‐ECN and Pd(PPh_3_)_4_ catalysts. Reproduced with permission.[qv: 3b] Copyright 2018, Nature Publishing Group. b) Catalytic performance of Co‐N_4_‐C SAC for oxidative C–C coupling reaction. Reproduced with permission.^95^ Copyright 2015, American Chemical Society. c) Schematic illustration of the synthesis of Pt‐ISA/NG catalyst and reaction model for hydrosilylation of 1‐octene. Reproduced with permission.^96^ Copyright 2018, American Chemical Society.

Pérez‐Ramírez and co‐workers successfully anchored Pd atoms onto exfoliated graphitic carbon nitride (Pd‐ECN), and a systematically comparison study demonstrates that the Pd SAC@ECN performed much better than the other catalysts with a TOF value of 549 h^−1^ and good stability, outperformed homogeneous Pd(PPh_3_)_4_ (34 h^−1^) and single‐site PdAc‐MPES/SiO_2_ (56 h^−1^), as shown in **Figure** 12a.[qv: 3b] There is no doubt that Pd‐ECN excels Pd complex both in activity and selectivity. In addition, the authors examined whether 2D structure benefits the catalytic performance by performing comparison experiment on Pd‐ECN and Pd SAC over mesoporous carbon nitride (Pd‐MCN). Amazingly, the Pd‐ECN is five times more active than Pd‐MCN (TOF: 97 h^−1^), demonstrating the advantages for employing 2D supports. To understand the excellent performance of Pd‐ECN, the reaction pathway was investigated with DFT calculations, which suggested that the barrier was low due to the dynamic coordination environment of Pd at different steps. This example offers an approach for designing new catalysts through modifying the coordinating sites of a given support.

To sum up, despite inspiring works have been accomplished, the current systems are still limited and the TOF values of SACs based on surface metal atoms are comparable to those of metal NPs and SAC@3D supports, and the intrinsic advantages of 2D materials such as high surface area and high electron mobility. Therefore, more systems need developing to make the best of 2D materials and come to a mild but highly efficient catalytic platform.

#### 5.1.4 Phenol Production from Benzene Oxidation

Apart from activating Si—H bonds, metal SACs@2D materials are also effective for catalyzing the breakage of C—H to form high‐value chemicals. In recent years, single metal atoms on 2D supports have found applications in direct oxidation of methane,[qv: 26b,97] benzene,^13^, ^48^, ^98^ and ethylbenzene^46^ to values products, which are receiving increasing attention.

**Figure 13:**
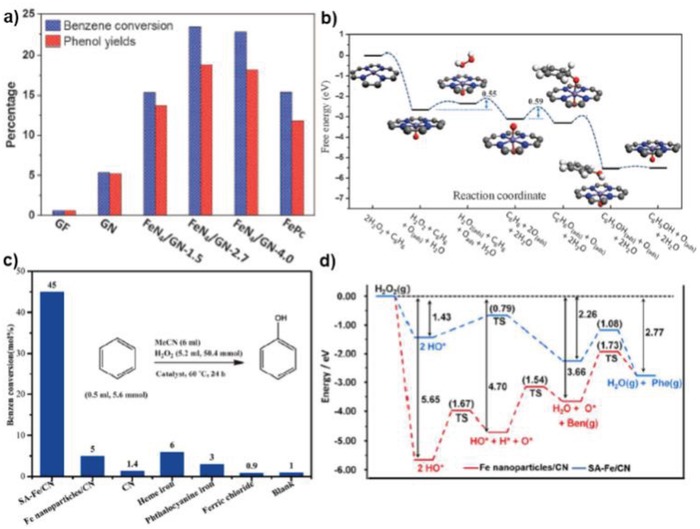
a) The performance of the direct oxidation of benzene to phenol by FeN_4_/GN samples; b) Free energy diagram of the oxidation of benzene to phenol on FeN_4_/NG. Reproduced with permission.^13^ Copyright 2015, AAAS. c,d) Catalytic performance of benzene oxidation over different Fe catalysts and the reaction mechanisms. Reproduced with permission.^48^ Copyright 2017, American Chemical Society.

Bao's group prepared a series of Fe SACs over N‐doped graphene with different Fe loadings, where Fe was connected with graphene through the FeN_4_ coordination.^13^ After optimization, the best Fe SAC, FeN_4_/GN‐2.7, efficiently catalyzed this reaction with the TOF reaching 84.7 h^−1^ at the initial stage. In **Figure** 13a, it is obvious that FeN_4_/GN‐2.7 outperformed the catalyst precursor FePc after 24 h in conversion (23.4% vs 15.4%) at similar selectivity (≈80%) and the whole reaction pathway was determined via DFT calculations that a hydrogen peroxide molecule detached easily from the Fe after forming a double bonds between Fe and O. Inspired by this work, Li's group further built up another Fe SAC system (SA‐Fe/NC) through pyrolysis of a polymer modified the metal precursor.^48^ As shown in Table 4 and Figure 13c, this SAC performed much better than corresponding metal precursor (FeCl_3_) and CN supported Fe NPs. The apparent turnover frequency (ATOF) during 24 h was estimated to be 2.9 h^−1^, which is still one of the best among Fe catalysts. Theoretical calculations were conducted for both Fe SAC and Fe NPs to explain the extraordinary catalytic activity of SA‐Fe/CN (Figure 13d). The key step in this reaction is the interaction between activated O species from H_2_O_2_ and benzene molecule. The transformation from the absorbed hydroxyl groups to activated O species is far easier on Fe SAC (energy barrier: 0.79 eV) than on Fe NPs (1.67 eV). What is more, the reaction of the activated O with benzene only needs to overcome a barrier of 1.08 eV on SA‐Fe/CN, while the energy barrier on Fe NP/CN is 1.73 eV. This explains why SA‐Fe/CN and Fe NP/CN behaved so different in this reaction. Although the 2D support is not directly involved in the catalytic cycle, it modifies the electronic structure of Fe atoms and stabilizes the singly dispersed species, making it outperform the metallic Fe NPs. Later, Li and co‐workers extended the Fe SAC system to a Co SAC supported on N‐doped carbon nanosheet (Co‐ISA/CNS), which was superior to all the Fe SACs (**Table** 5).^96^ Mechanistic studies illustrated that benzene oxidation to phenol on Co SAC followed a similar route over SA‐Fe/CN, and the main factors for the excellent performance might be the Co–N coordination environment and Co single‐atom identity in Co‐ISA/CNS. All these examples demonstrate the crucial role of the N‐doped graphene in promoting the production of phenol from benzene. This suggests that the rational introduction of the electronegative heteroatoms to 2D materials not only strengthens the metal–support interaction, but also modulates the electronic states of metal atoms subtly, which is useful for designing SACs for other applications. In the case of 3D materials, however, controllable introduction of heteroatoms with well‐defined catalytic centers might not be that easy, and it is difficult to modulate the electronic properties of the supports prominently due to the heterogeneous structures of 3D supports. Furthermore, the adsorption of benzene on single metal atoms indicates the potential for applying metal SACs@2D materials for highly efficient phenol hydrogenation to cyclohexanone, which is more promising in industry.

**Table 5:** List of metal SACs@2D supports for benzene oxidation reaction with 30% H_2_O_2_ as the oxidant

No	Catalyst	S/C ratio	*T* [°C]	ATOF^a)^ [h^−1^]	S to phenol [%]	Refs.
1	GN	–	25	–	97.1	13
2	FeN_4_/GN‐1.5	335	25	2.1	89.4	13
3	FeN_4_/GN‐2.7	186	25	84.7	94.3	13
4	FeN_4_/GN‐2.7	186	25	1.8	80.0	13
5	FeN_4_/GN‐4.0	126	25	1.2	79.8	13
6	FeN_4_/GN‐1.5	335	0	1.1	87.6	13
7	FeN_4_/GN‐2.7	186	0	0.71	89.8	13
8	SA‐Fe/CN	697	60	13.1	94	48
9	Co‐ISA/CNS	884	25	150	93.3	96
10	Co‐ISA/CNS	884	25	6.3	89.7	96

^a)^ ATOF means the number of converted reactants per metal atom per hour on average.

#### 5.1.5 NH_3_ Synthesis via N_2_ Reduction

Ammonia gas (NH_3_) belongs to one of the most demanding chemicals in industry, but current Haber–Bosch process requires harsh conditions and causes a waste of energy. Thus, synthesizing NH_3_ with an energy‐efficient approach has attracted tremendous attempts, among which electrocatalysis and photocatalysis over metal SACs exhibited promising performance for this reaction.^99^

Xie's group loaded atomically dispersed Cu species on ultrathin polymer carbon nitride (p‐CN) nanosheets, and the Cu/p‐CN exhibited exciting performance in photoilluminated synthesis of NH_3_ under ambient conditions. In the Cu SAC, Cu atom adopted a threefold coordination mode with N atoms, resulting in the single valence electron of the coordinated N atom delocalized even isolated from the π conjugated electron cloud due to the charge density difference. Evidenced by ESR results, isolated electrons are more readily activated for participating in the photocatalytic reactions. The catalytic results showed that the photocatalytic ammonia synthesis activity of Cu/p‐CN SAC was improved by seven times compared with that of metal‐free p‐CN, reaching 186 µmol g^−1^ h^−1^ under visible light irradiation. These findings demonstrated the manipulation of lone‐pair electrons to realize the effective isolation of conjugated valence electrons is a facile strategy for achieving exciting photocatalytic performance.

**Figure 14:**
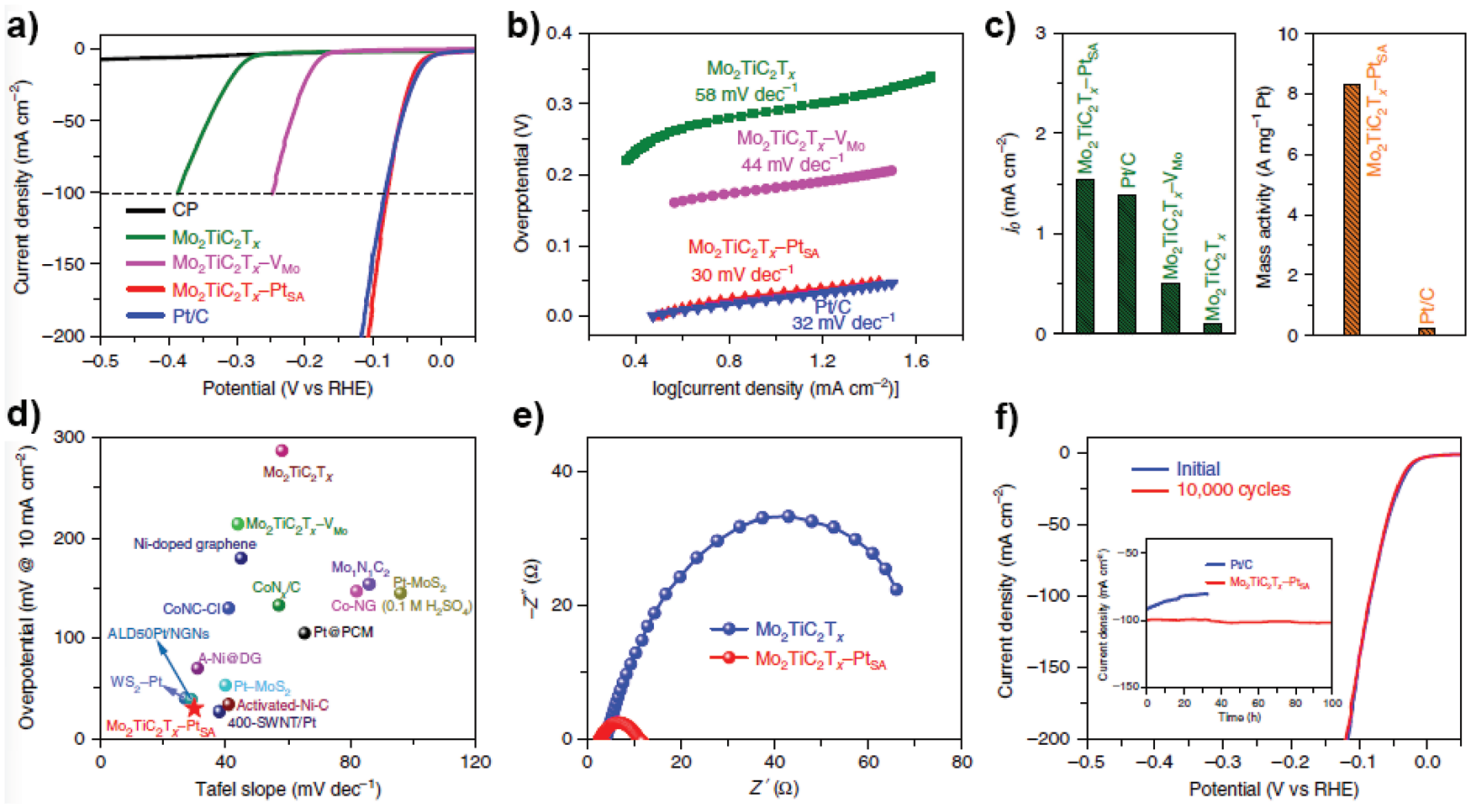
Electrocatalytic performance for Mo_2_TiC_2_T*_x_*–Pt_SA_ and reference HER catalysts. a) HER polarization curves of carbon paper (CP), Mo_2_TiC_2_T_*x*_,Mo_2_TiC_2_T_*x*_–V_Mo_, Mo_2_TiC_2_T_*x*_–Pt_SA_ and Pt/C (40%). b) Corresponding Tafel slope derived from a. c) Exchange current densities of the catalysts, and the mass activity of state‐of‐the‐art Pt/C and Mo_2_TiC_2_T_*x*_–Pt_SA_. d) Comparison of Tafel slope and overpotential (10 mA cm^−2^) for various single‐atom or Pt‐based HER catalysts in 0.5 m H_2_SO_4_ solution. e) EIS Nyquist plots of Mo_2_TiC_2_T_*x*_–Pt_SA_ and Mo_2_TiC_2_T_*x*_ catalysts. f) Stability test of Mo_2_TiC_2_T_*x*_–Pt_SA_ through potential cycling, before and after 10 000 cycles. Reproduced with permission.^29^ Copyright 2018, Nature Publishing Group.

**Figure 15:**
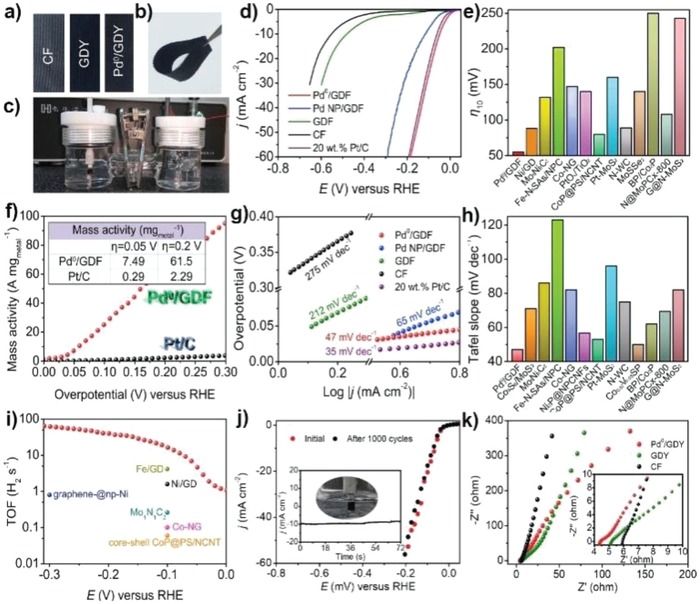
HER Performances of Pd^0^/GDY. a) Photographs of Pd^0^/GDY, GDY, and CF, which was used as the working electrode for the HER test. b) Photograph displaying the flexibility of Pd^0^/GDY. c) Photograph of the established three‐electrode system. d) Polarization curves of Pd^0^/GDY, Pd NP/GDY, GDY, CF, and Pt/C. e) Overpotentials at 10 mA cm^2^ of other recent ACs and several bulk catalysts. f) Mass activity of Pd^0^/GDY and Pt/C (inset: mass activity collected at overpotentials of 0.05 and 0.2 V). g) Corresponding Tafel slope of the catalysts in a). h) Tafel slopes of other recent ACs and several bulk catalysts. i) TOF values of Pd^0^/GDY together with those of several recent ACs and bulk catalysts. j) Polarization curves of Pd^0^/GDY before and after 1000 cycle tests. k) Nyquist plots of the catalysts. Reproduced with permission.^30^ Copyright 2019, Cell Press.

In another study by Wang et al., Au_1_/g‐C_3_N_4_ was prepared and utilized for synthesizing NH_3_ under mild conditions via electrochemical approach (**Figure** 14).[qv: 99a] In comparison with Au NPs/g‐C_3_N_4_ in **Figure** 15b, Au_1_/g‐C_3_N_4_ showed higher NH_4_^+^Faradaic efficiencies at the applied potential from −0.1 to −0.5 V. The Faradaic efficiency over Au SAC reached as high as of NH_4_^+^ of 11.1%, among the best catalysts for this reaction under comparable conditions. Benefiting from efficient atom utilization, an NH_4_^+^ yield rate of 1305 µg h^−1^ mg Au^−1^ has been reached, which is roughly 22.5 times as high as that by supported Au nanoparticles (Figure 11c). Further, this study also proved that NH^4+^ can be electrochemically produced directly from N_2_ and H_2_ with energy utilization rate of 4.02 mmol kJ^−1^ over the Au SAC, which offers a possible way to replace the Haber‐Bosch process with energy efficient and environmentally friendly electrochemical approaches.

#### 5.1.6 Others

In addition to the above‐mentioned chemicals, metal SACs@2D materials were also reported to catalyze the production of aldehyde via hydroformylation reaction (Rh atoms on CoO nanosheet),^65^ H_2_O_2_ using electrochemical process over Pt–S_4_–C,[qv: 6b] saturated benzyl esters through selective hydrogenation reaction without inducing hydrolysis (Ru on Pd nanoribbons). Based on the discussion above, it is obvious that 2D supports offer SACs a large number of well‐defined catalytically active sites, M–N–C or M–O, throughout the whole support surface. Due to the uniform structures of 2D materials, the dopant of metal atoms also alters the electronic properties of supports drastically. The special interaction between metal atoms and 2D supports contributes to the attractive catalytic performance in chemical productions.

Despite the fast development of metal SACs on chemical production, the variety of chemicals produced over SACs is not comparable to that over NP catalysts, and 3D material‐supported SACs are more widely utilized than SACs@2D materials. It is noteworthy that the efficient production of more chemicals is proved approachable on 2D material‐supported metal SACs by theoretical calculations, which shed light on the direction for future catalyst design and catalytic applications. Taking ammonia synthesis as an example, Ma and co‐workers employed DFT calculations to show that Fe supported on phosphorene could promote the N_2_ fixation process via three possible pathways, which is due to the coordinating effect between Fe and phosphorus atoms.^100^ 3D supports excel 2D supports due to the easy synthesis and more varieties. As discussed above, almost all the active centers can be identified over metal SACs@2D materials, and theoretical studies are more accurate than those on simplified nonuniform 3D supports. Theoretical calculation‐guided chemical production is promising and practical for SACs@2D supports, and more efforts are worth devoting to.

### 5.2 Application of SAC on 2D Materials for Energy Field

Developing efficient energy conversion processes for substituting conversional energy sources with sustainable and clean energy is highly demanding due to overconsumption of fossil fuels and the consequent environmental issues. Thanks to the unique electronic structures of 2D supports, the combination of metal species and 2D supports is capable for catalyzing a series of electrochemical and photochemical processes. Among these processes, there are many reactions dealing with the urgent energy issues, such as HER for producing H_2_, ORR, methanol oxidation reaction (MOR), etc. Herein, the applications of metal SACs on 2D materials in fuel production through hydrodeoxygenation (HDO) reaction, ORR, H_2_ production from HER and NH_3_BH_3_ hydrolysis and OER.

#### 5.2.1 Aromatic Fuels Production via HDO Reactions

Hydrodeoxygenation and hydrogenation are important processes for converting biomass compounds and their derivative oxygenated chemicals to high value‐added chemicals and fuels. Tremendous effect has been put to promote the development of highly effective and ecofriendly catalysts for such reactions. Traditional noble metal NP catalysts are efficient, but suffer from environmental issues. Non‐noble metals usually work under very harsh conditions, inhibiting their practical applications.

**Figure 16:**
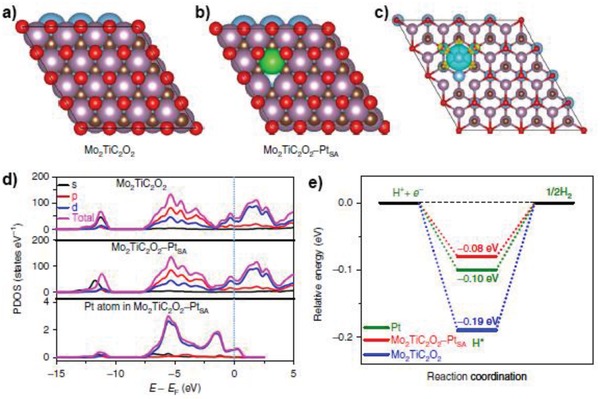
Top view of the slab models used to describe a) Mo_2_TiC_2_O_2_ and b) Mo_2_TiC_2_O_2_–Pt_SA_. Atoms in blue, purple, green, brown, and red represent Ti, Mo, Pt, C, and O, respectively. c) Calculated charge density distribution differences between Mo_2_TiC_2_O_2_ and Mo_2_TiC_2_O_2_–Pt_SA_.The isosurface value is 0.01 e A^−3^ to better plot the differences. d) Calculated PDOS of Mo_2_TiC_2_O_2_ and Mo_2_TiC_2_O_2_–Pt_SA_, with aligned Fermi level. e) Calculated free energy profiles of HER at the equilibrium potential for Mo_2_TiC_2_O_2_, Mo_2_TiC_2_O_2_–Pt_SA_ and Pt/C. Reproduced with permission.^29^ Copyright 2018, Nature Publishing Group.

**Figure 17:**
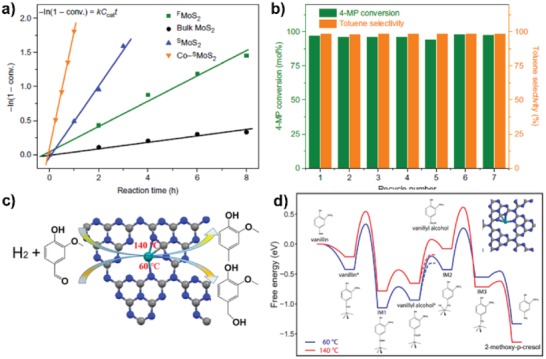
a,b) Superior activity and stability of Co–^S^MoS_2_. Reproduced with permission.^83^ Copyright 2017, Nature Publishing Group. c,d) Reaction pathway of vanillin hydrogenation and HDO over Ru_1_/g‐C_3_N_4_. Reproduced with permission.[qv: 3c] Copyright 2018, American Chemical Society.

Conventional Mo‐based catalysts are active for breaking the C—O bond usually when the reaction temperature increases to around 300 °C. It is found that the monolayer MoS_2_ support is 2.8 times more active in HDO of 4‐methyphenol to toluene than few‐layer MoS_2_, achieving a conversion of 98.7% and selectivity to toluene of 83.1% after reaction at 300 °C for 5 h. Inspired by this point, Tsang and co‐workers synthesized a Co SAC on monolayer MoS_2_, where Co atoms bonded to the sulfur vacancies on the basal planes, and examined its catalytic performance on.^83^ Encouragingly, the addition of Co atoms dramatically increased the performance with conversion reaching 83.6% (selectivity towards toluene is 99.2%) after only 1 h reaction at the identical conditions. As shown in **Figure** 16a, this Co SAC exhibited extraordinary activity compared to pure MoS_2_. Further, this Co–^s^MoS_2_ SAC successfully converted 97.6% of 4‐methylphenol, yielding 96.0% of toluene, which reduced the active temperature by 120 °C with admirable stability (**Figure** 17b).The interaction between atomically dispersed Co species and monolayer MoS_2_via covalent bonds enhances the stability of both Co atoms and the support, which overcomes the desulfurization issue encountered by conversional CoMoS_2_ catalysts. This suggests that 2D structure is important for creating highly efficient sites.

Further, Tian et al. fabricated a Ru SAC on mesoporous C_3_N_4_ (Ru_1_/mpg‐C_3_N_4_), which shows excellent temperature controlled hydrogenation and HDO performance.[qv: 3c] As depicted in Figure 17c,d, HDO requires higher temperature and 100% selectivity toward the HDO product (2‐methoxyl‐p‐cresol) was realized at 140 °C. Even compared with the benchmark Pd‐based catalysts, this Ru SAC is among the best ones with a TOF of 330 h^−1^. When performing this reaction below 60 °C, the sole product was hydrogenated one (vanillyl alcohol). Theoretical calculations reveal that Ru atoms were dynamic, and that active sites were induced by adsorption of reactants.

Up to now, HDO reactions over metal SACs are scarcely explored, which is probably due to the relatively severe conditions required (high temperatures and H_2_ pressures), which will result in aggregation of metal atoms. Investigations on the catalytic mechanisms are not thoroughly conducted, and thus conclusive roles of 2D supports remain undecided. Appropriate SACs@2D material nanoplatforms requires developing for HDO routes.

#### 5.2.2 Oxygen Reduction Reaction

The electrocatalytic ORR plays a significant role in electrochemical devices including metal–air batteries and fuel cells. ORR can proceed in both alkaline and acidic media, during which the reduction might adopt two‐electron or four‐electron mode. The four‐electron process excels two‐electron process in efficiency and prevents forming of H_2_O_2_ that might cause damage to the membrane and ionomer. Heteroatom‐doped 2D carbon materials are good catalysts for electrocatalytic ORR reactions. Recently, it has been proved that metal SACs@2D carbon materials prominently enhance the ORR performance.[qv: 35,55,57,58,66b,101]

**Figure 18:**
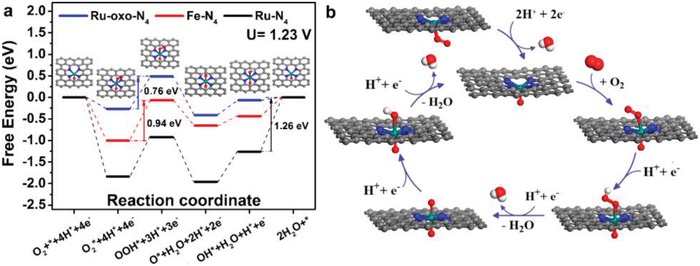
a) Free‐energy diagram of the ORR on selected nitrogen‐coordinated metal moieties embedded on graphene sheets. b) Proposed reaction scheme of the associative mechanism for the ORR on Ru–oxo–N_4_ moiety in acidic medium. Reproduced with permission.[qv: 101a] Copyright 2017, American Chemical Society.

The exceptional ORR activity with single metal atom dopants might be attributed to the moderate adsorption strength of key ORR intermediates on metal centers, which would influence the reaction pathway. For example, S‐doped graphene has strong affinity toward Pt atoms, which stabilizes Pt atoms through a Pt–S_4_ bonding. Choi and co‐workers tested ORR over this Pt‐S4‐C SAC, and found that this Pt SAC preferentially catalyzed a two‐electron oxygen reduction pathway for H_2_O_2_ production rather than the four‐electron oxygen reduction pathway on traditional Pt catalysts, which is consistent with the theoretical calculations.^102^ Liu's group reported another Pt SAC with N‐doped carbon as the support (Pt1–N/BP), which showed good activity for ORR and admirable tolerance for methanol/CO poisoning in acidic media.^103^ Zhang et al. anchored atomically dispersed Ru on N‐doped graphene (Ru/N‐doped G), which exhibits excellent four‐electron ORR activity, offering onset and half‐wave potentials of 0.89 and 0.75 V, respectively, together with better durability and tolerance toward methanol and CO poisoning than commercial Pt/C catalysts. DFT calculations further indicated that catalytic activity of Ru‐N/G in ORR originated from the Ru‐oxo‐N_4_ moieties instead of Ru‐N_4_ during the oxidative electrocatalytic condition, which shed light on future designs of powerful catalysts for ORR in acid media (**Figure** 18).[qv: 101a] In addition to noble metals, N‐doped carbon supported transitional metal SACs are also active in ORR processes.^55^, ^70^, ^104^ It is worth noting that these studies are based on catalytic active supports, so the contribution to the overall ORR performance is not easy for clarifying. An unresolved question is whether the ligands from the supports participates in the ORR cycle or just alters the charge states of metal atoms. Theoretical results provide useful guides, but experimental evidences are more favorable and convincing. Control studies with inactive supports might be interesting to illustrate the unique reactivity of SACs. Otherwise, it is hard to conclude the major contributor.

#### 5.2.3 Hydrogen Production

*H_2_ Release from Thermal Decomposition of Hydrogen Storage Materials*: Hydrogen is regarded as one type of next‐generation clean energy fuels, which have been used to replace the fast depleting fossil fuels. H_2_ production by catalytic decomposition of hydrogen storage materials is an economic, efficient, and convenient way to utilize hydrogen energy in practice. Ammonia‐borane (NH_3_BH_3_), formic acid (HCOOH), hydrous hydrazine (N_2_H_4_·H_2_O), and methanol are typical H_2_ storage materials, so developing highly efficient catalysts for releasing H_2_ are urgent issues in hydrogen economy. In the past few years, 2D carbon material‐supported atomically dispersed metal catalysts have exhibited great performance for catalyzing the hydrolysis of ammonia‐borane and decomposition of formic acid.[qv: 11b]

**Figure 19:**
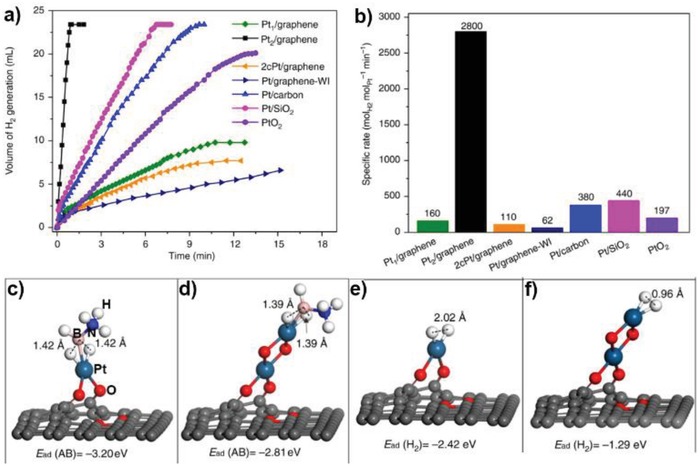
a,b) Catalytic activities of various Pt catalysts in AB hydrolysis. c–f) The local configurations for AB adsorption on reduced Pt_1_/graphene and Pt_2_/graphene. Reproduced with permission.[qv: 11b] Copyright 2017, Nature Publishing Group.

Lu's research group successfully applied a graphene supported Pt_2_ dimers, where Pt atoms are bridged with the Pt–O_2_–Pt configuration, to efficiently catalyze the hydrolysis ammonia borane (AB).[qv: 11b] Pt_2_/graphene was prepared on the basis of Pt_1_/graphene catalyst through the ALD method. As shown in **Figure** 19, Pt dimers and Pt_2_/graphene had a much better performance than the Pt_1_/graphene and all Pt NP catalysts, with a specific rate of as high as 2800 mol_H2_ mol_Pt_^−1^ min^−1^ at ambient condition, which is around 17 times of the value on Pt_1_/graphene SAC and 44 times more active than the same graphene‐supported nanoparticles. The adsorption energy of AB on Pt_2_/graphene indicated weaker adsorption of AB on Pt dimers, which alleviates the problem of boron poisoning compared to Pt_1_/graphene and Pt NPs. Besides, Figure 19e shows that the produced H_2_ molecule strongly absorbed on Pt_1_ and dissociated with an H–H distance of 2.02 Å. In contrast, H_2_ did not dissociate on Pt dimers and the adsorption was much weaker, indicating the H_2_ might leave Pt atoms easily after formation. It is obvious that the support effect was not included for mechanistic studies, which does not reflect the real case. The graphene support has been oxidized prior to loading metal species, so a more accurate calculations should be based on GO. The applications for thermal H_2_ production over SACs@2D supports are far from satisfactory and fall behind SACs@3D supports. A main reason might be that for this application good performance instead of deep insights into the mechanisms is the most important.

*Eletrocatalytic Hydrogen Evolution Reaction*: Currently, evolution of H_2_ from the water splitting reaction is one of the most investigated approaches, where electrochemical and photochemical techniques are usually employed. The water splitting reaction is generally divided into two half‐reactions, with HER processing at the cathode (2H^+^ + 2*e* ^−^ → H_2_) and OER (H_2_O → 2H^+^ + 1/2O_2_+ 2*e* ^−^) taking place at the anode. A complete water splitting reaction will decompose two water molecules into two H_2_ and one O_2_. Thermodynamically, the minimum potential for completing the overall water splitting reaction is 1.23 V. In recent years, HER catalyzed by supported metal SACs, especially 2D materials (MXene, graphdiyne, graphitic carbon materials, and MoS_2_) supported metal SACs are widely investigated, as listed in **Table** 6. As the HER of SACs supported on MoS_2_, g‐C_3_N_4_ and graphitic carbon have been reviewed in detail previously,^15^, ^105^ this section is focused on the novel supports.

**Table 6:** Summary of selected metal SACs@2D supports for electrocatalytic HER in 0.5 m H_2_SO_4_

Metal	Support	Working electrode	Overpotential (10 mA cm^−2^) [mV]	Tafel slope [mV dec^−1^]	Refs.
Pt	MXene	Carbon paper	30	30	29
Pt	Fe–N–C	Glassy carbon electrode	60	32	70
Pt	N‐doped graphene	Glassy carbon electrode	50	29	38
Pt	MoS_2_	Glassy carbon electrode	145	96	[qv: 17a]
Pd	Graphdiyne	Pd^0^/graphdiyne	55	47	30
Pd	MoS_2_	Glassy carbon electrode	78	62–80	106
Fe	N‐doped graphene	Glassy carbon electrode	202	123	107
Fe	Graphdiyne	Graphite plate	66	38	73
Ni	Graphdiyne	Graphite plate	88	45	73
Ni	Graphene	Ni substrates	50	45	71
Co	N‐doped carbon	Glassy carbon disk	133	57	43
Mo	N‐doped carbon	Glassy carbon electrode	154	86	52

**Figure 20:**
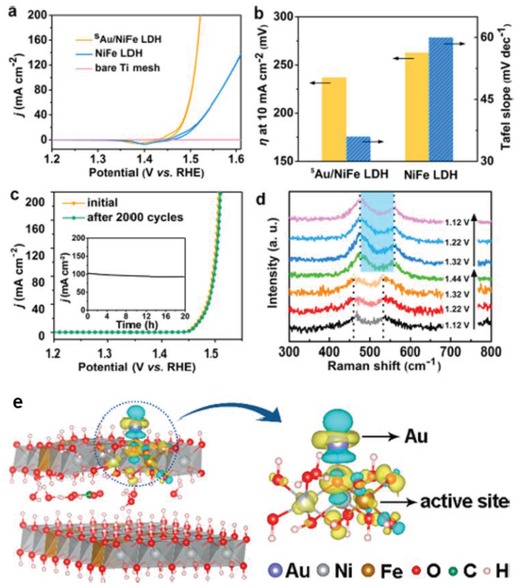
a) CV curves of ^s^Au/NiFeLDH, pure NiFe LDH and bare Ti mesh in 1 m KOH. b) Overpotential at 10 mA cm^−2^ and Tafel slope for ^s^Au/NiFe LDH and pure NiFe LDH. c) Polarization curves of ^s^Au/NiFe LDH before and after 2000 cycles. d) Raman spectra of ^s^Au/NiFe LDH at different potentials in a CV cycle. e) Differential charge densities of NiFe LDH with and without Au atom when one O atom is adsorbed on the Fe site. Reproduced with permission.^112^ Copyright 2018, American Chemical Society.

Wang and co‐workers designed a Pt SAC by immobilizing single Pt atoms on the Mo vacancies of a double transition metal MXene (Mo_2_TiC_2_T*_x_*), where each Pt atom coordinated with two C atoms on the MXene.^29^ As depicted in Figure 14a, this Pt SAC showed similar kinetic behavior for HER to the state‐of‐art commercial Pt/C with a Tafel slope of 30 mV per dec toward HER and exhibited increased catalytic activity (30, 77, and 104 mV at 10100, and 200 mA cm^−2^, respectively) without appreciable deactivation for 10 000 cycles. Besides the high exchange current density (Figure 14c), the mass activity of Pt SAC is 40, which is five times of the value over commercial Pt/C (8.3 vs 0.21 A mg^‐1^). The amazing performance with low Tafel slope and overpotential also outperformed other previously reported Pt SACs (Figure 14d). **Figure** 20e reveals that the resistance for charge transfer over Pt/MXene SAC was much lower than the support, which might be the reason for the highly efficient Faradaic process and outstanding HER performance. To understand how the 2D support interacts with Pt atoms based on the charge density distribution in Figure 16c, the incorporation of Pt atoms reconstructs the electronic structure of the MXene, which induces high electron density around Pt sites. The projected density of states (PDOS) results in Figure 16d suggests that Pt SAC shows higher occupied states than the bare MXene near the Fermi level, which promotes the electron transfer and induces greater conductivity. Comparison between the results of Pt/MXene and MXene suggests that Pt atom itself could efficiently increase the d‐electron occupation around the Fermi level, which is associated with high catalytic performance. Furthermore, the Gibbs free energy (Δ *G*_H*_) for hydrogen adsorption was studied to elucidate the excellent catalytic activity. In general, HER process contains three steps in acidic solution, which are adsorption of H^+^ on catalytic centers, proton reduction by electron to form of adsorbed hydrogen species H*, and H_2_ release. It has been generally accepted that when the optimal Δ *G*_H*_ for hydrogen adsorption is close to zero, the catalyst is good for HER, which is a major descriptor for various HER catalysts. Figure 16e shows that the Δ *G*_H_* of Pt SAC is only ‐0.08 eV, which is lower than that of state of Pt/C (‐0.10 eV) and the MXene support (‐0.19 eV), confirming that this MXene supported Pt SAC is a good HER catalyst. This proved that the H_2_ adsorption on Pt atoms SAC is weaker than on Pt/C and MXene, resulting in faster production and release of H_2_.

Another novel 2D materials, GDY, has also received great attention for fabricating metal SACs.^108^ GDY is a carbon allotrope whose carbon atoms are sp^2^‐hybridized in aromatic rings and connected with sp‐hybridized carbon atoms of diacetylenic groups. The coexistence of different hybridization modes in GDY indicates that the rotation of the π/π* orbitals can happen in any direction perpendicular to the C≡C bond, which makes GDY a great support for confining metal atoms via strong electronic interaction. Yu et al. reported a Pd SAC loaded on ultrathin GDY nanosheet and its direct application in HER cathode.^30^ After convincingly confirmed the structure of Pd SAC (Pd^0^/GDY), the catalytic performance for HER was examined together with other catalysts (Figure 15). As shown in Figure 15, Pd SAC exhibited an overpotential of 55 mV, which is lower than all the other catalysts. Besides, Pd SAC behaved much better than the state of art commercial Pt/C catalyst in both mass activity (61.5 A mg_metal_^−1^ vs 2.29 A mg_metal_^−1^) and TOF (16.7 s^−1^ vs 11.5 s^−1^) under identical conditions. The deduced value of exchange current density showed is 0.282 mA cm^−2^, significantly higher than most of previously reported values. This indicates that loading of 0.2 wt% Pd drastically enhanced the HER rate. Pd^0^/GDY demonstrated its great stability after surviving 72 h of continuous HER tests without detectable decay in activity stability. The exceptional electrocatalytic performance of Pd^0^/GDY was attributed to the unique electronic and geometric structure, which resulted in a synergistic effect.

To sum up, SACs on traditional and emerging 2D supports have great potential for electrocatalytic HER processes. How to apply them into practical processes with inspiring performance is highly needed. As shown in Table 6, more SAC@2D material nanoplatforms require exploring, which indicates the vast potential for HER studies.

**Figure 21:**
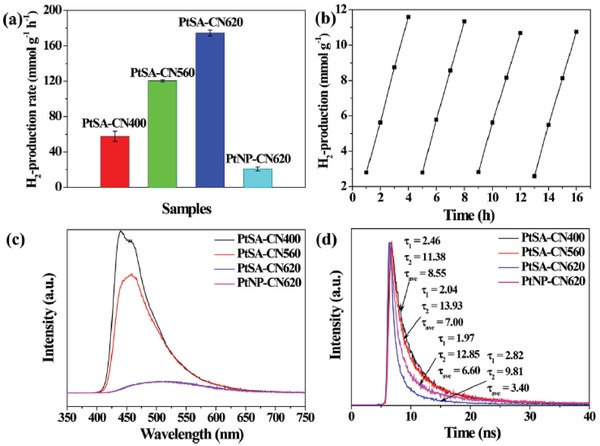
a) The H_2_ evolution rates of PtSA–CN400, PtSA–CN560, PtSA–CN620, and PtNP–CN620 normalized to the mass of Pt cocatalyst. b) Long‐term stability test, c) the steady‐state and time‐resolved photoluminescence spectra of different catalysts, and d) the cycling H_2_ production activity of PtSA–CN620. Reproduced with permission.^72^ Copyright 2019, Elsevier.

*Photocatalytic Water‐Splitting Reaction*: Photocatalytic HER is also a common practice for producing H_2_. In recent years, metal SACs on g‐C_3_N_4_ have exhibited superior photocataytic activity in HER.^72^, ^109^ For instance, Xie and co‐workers reported a Pt SAC on g‐C_3_N_4,_ which dramatically enhanced HER activity of the bare support. The Pt SAC efficiently H_2_at the rate of 318 µmol h^−1^, which was almost 51 time of the support.^63^ Meanwhile, the authors examined the catalytic performance on the same carbon nitride supported Pt NPs with different sizes, with Pt SAC behaved best. In terms of explanation for the superior activity on Pt SAC, the authors attributed the reason to the intrinsic modulation of the support surface trap states caused by the incorporation of Pt atoms along with more active sites. Zhou et al. synthesized a series of graphitic carbon nitride that were calcined at different temperatures and loaded Pt atoms on all the supports for photocatalytic HER.^72^ As depicted in **Figure** 21, all the Pt SACs performed better than the Pt NPs. Comparing PtSA–CN620 and PtNP–CN620, the mass activity over the SAC was more than eight times of that over Pt NP. Compared to PtSA–CN400 and PtSA–CN560 which began to form Pt NPs during the reaction, the best Pt SAC, PtSA–CN620 exhibited stability without Pt aggregation or deactivation in a 16 h run. Figure 21c shows that the steady‐state photoluminescence (PL) peak of PtSA–CN620 at 515 nm was much weaker than the other two SACs, indicating longer lifetime for the photogenerated carrier in PtSA–CN620. Further, time‐resolved PL demonstrated that the charge transfer in PtSA–CN620 was faster than the other SACs, which was ascribed to the synergistic effect of single Pt atoms and N vacancies. The reactive metal–support interaction in PtSA–CN620 originating from the enriched N vacancies in the support helped to boost the HER reaction and enhance the stability.

Furthermore, Xiong and co‐workers discovered g‐C_3_N_4_ supported Pt cation (Pt^2+^) where Pt cations were confined in the micropore of carbon nitride via bonding Pt—N bonding, also exhibited better HER activity (605 µmol g^−1^ h^−1^) than the bare carbon nitride.^45^ Unfortunately, experimental results indicated that isolated Pt cations could not function as a cocatalyst for HER. To shed light on this issue, DFT calculations on the DOS the Pt SAC and pure support were conducted, the result of which suggested that the excited electrons in Pt cations below the Fermi level were transferred to the orbital of g‐C_3_N_4_ above the Fermi level, broadening the light absorption of the support. The so‐called metal‐to‐ligand charge transfer is responsible for the high HER activity. Besides, noble metals, supported transition metals have also been explore for photocatalysis. For example, Wei's research group anchored single Co atoms on phosphorus doped g‐C_3_N_4_ via a Co‐P_4_ bonding, which showed excellent photocatalytic HER activity with a H_2_ evolution rate of 410.3 µmol h^−1^ g^−1^ under visible light and quantum efficiency up to 2.2% at 500 nm.^53^ The well‐distributed Co–P_4_ sites promotes the effective charge‐hole separation and transfer, and thus benefits both HER and OER. This work served as an example exploiting the potential of ligated single atom site as an effective catalyst for photocatalytic water splitting reaction. Similarly, another carbon nitride supported cobalt SAC with Co atoms forming Co‐N_4_ site was also reported effective for HER, with the H_2_ evolution rate reaching 10.8 µmol h^−1^ g^−1^, which was 12 times of pure carbon nitride.^110^ Unlike the Co–P_4_ structure discussed above, the photogenerated e^−^ transferred from the electron‐donating N atoms to Co atoms, resulting in activity for H^+^ reduction. Thus, it makes sense to employ non‐noble metal atoms to create catalytically active sites for enhancing the separation of photogenerated electron−hole pairs. All the cases discussed above involved both metal atoms and the supports, which demonstrates the great potential of SACs@2D for photocatalysis.

Generally speaking, the photocatalytic activity is from the support with specific bandgap, and the doping of metal atoms onto the supports induces the rearrangement of the electrons on the supports via charge transfer. Most of the supports applied to photocatalysis are 3D materials such as TiO_2_ and GaP with excellent bandgap and long history of research, while 2D materials are less actively investigated for photocatalysis. The addition of metal atoms would alter the whole electronic environment or coordination modes of the supports, which in turn modulates the metal charges. Therefore, to make the best of synergistic effects of 2D materials and single metal atoms, explorations on their photocatalytic performance and more importantly, mechanistic studies cannot be ignored.

*Oxygen Evolution Reaction*: Oxygen evolution reaction (OER) containing 4 steps H^+^ coupled charge transfer makes the kinetics slow, resulting in more energy consumption for stepping over the barrier to produce O_2_. Thus, it is more challenging to design an efficient OER catalyst than HER catalyst.^111^ Recently, metal SACs on 2D materials have been investigated for OER electrocatalysis.

NiFe‐LDHs are regarded good supports for electrocatalysis due to the tunable chemical composition, and LDH supported atomically dispersed metal species have been studied for OER both experimentally and theoretically. Zhang et al. obtained an Au SAC on NiFe LDH (^s^Au/NiFe LDH) using an electrodeposition approach.^112^ Prior to experimental studies, the authored performed theoretical calculations to elucidate that Fe sites were the catalytic active sites for OER and that the introduction of Au atoms to the LDH decreases the overpotential of the support. The OER activity and stability of LDH supported Au SAC was examined and the results are shown in Figure 20a–c. At 10 mA cm^−2^, the overpotentional for the ^s^Au/NiFe LDH and NiFe LDH were found to be 237 and 263 mV, respectively (Figure 20). Raman spectra in Figure 20d suggested that the transformation of NiFe LDHs to oxyhydroxides occurred under the OER potential, which consolidated the theoretical findings that the oxyhydroxide formed in situ functioned as the catalytic centers for OER (Figure 20e). Au atoms in this process assist in the electron transfer to the LDH in the catalyst, which changed the distribution of the surface charge and enhanced the OER performance.

**Figure 22:**
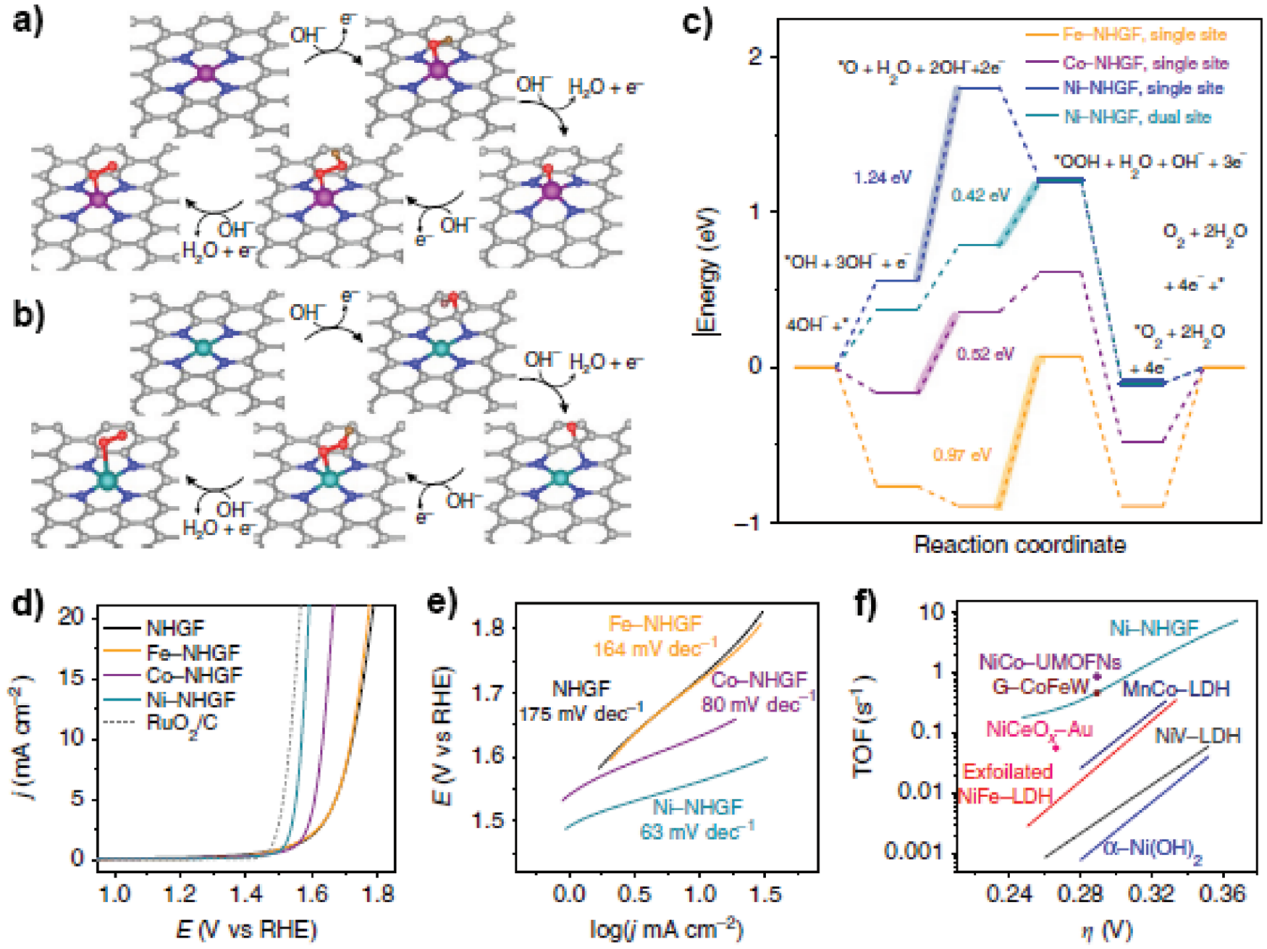
Catalytic OER activity evaluation by a–c) DFT simulations and d–f) electrochemical measurements. Reproduced with permission.^113^ Copyright 2018, Nature Publishing Group.

Besides LDH, N‐doped carbon and carbon nitride are the most utilized 2D supports for stabilizing metal SACs when catalyzing electrocatalytic OER (**Table** 7). Huang's research group constructed atomically dispersed metal‐N_4_‐C_4_sites on N‐doped holey graphene frameworks (M‐NHGF, M = Ni, Co, Fe) by thermal annealing treatment of graphene supported metal precursors with NH_3_.^113^ The M‐N_4_‐C_4_ site was demonstrated to the active center for OER process. As shown in **Figure** 22, Ni‐NHGF performed best among the three SACs, whereas Fe‐NHGF was the least active SAC. At a current density of 10 mA cm^−2^, the overpotential for Ni‐NHGF was measured to be 331 mV, while the values for Co‐NHGF and Fe‐NHGF were 402 and 488 mV, respectively. Furthermore, the Tafel slope results follow the same sequence: Ni‐NHGF (63 mV dec^−1^) < Co‐NHGF (80 mV dec^−1^) < Fe‐NHGF (164 mV dec^−1^). The analyses of TOF values for these SACs directly showed that the Ni SAC was among the best OER catalysts. Based on DFT calculation, two reaction mechanisms were proposed (Figure 22a,b). Theoretical studies showed that the involvement of C in OER process has direct relationship with electron number in d orbitals. In Fe (*N*_d_ = 6) and Co (*N*_d_ = 7) SACs, the intermediates were found to preferentially bond at the metal site rather than the C site, and thus OER followed the single‐site pathway.

**Table 7:** Summary of selected metal SACs@2D carbon materials for electrocatalytic OER

Metal	Support	Electrolytes	Overpotential (10 mA cm^−2^) [mV]	Tafel slope [mV dec^−1^]	Refs.
Fe	N‐doped graphene	1 m KOH	488	164	113
Co	N‐doped graphene	1 m KOH	402	80	113
Ni	N‐doped graphene	1 m KOH	301	63	113
NiFe	g‐C_3_N_4_	1 m KOH	326	67	114
Pt	Fe‐N‐C	0.1 m KOH	310	62	70
Ni	g‐C_3_N_4_	1 m KOH		60	49
Au	g‐C_3_N_4_	0.1 m KOH	450	112	[qv: 66b]

On the other hand, Ni atom contains 8 d electrons and O* and OH* were found to favor the carbon site, whereas the OOH* preferred to adsorb on the metal site. Compared with the single‐site mechanism, the dual‐site mechanisms helped decrease the energy barrier drastically. This dual‐site mechanism involving both metal species and the 2D support offers guidance for designing highly efficient metal SACs in the future. Single Ni atoms were also stabilized on the defects of N‐free graphene, which exhibited an even better performance in alkaline media than that of the Ni‐NHGF catalyst mentioned above.^10^ An overpotential of 270 mV was obtained at the current density of10 mA cm^−2^, outperforming that of the state‐of art IrO_2_. First‐principle calculations indicated that the defects dramatically affect the charge state of Ni, and the optimized structure for OER was Ni immobilized on the di‐vacancy defect of graphene. Liu et al. fabricated an Au SAC where Au_1_^+^ cation was immobilized on g‐C_3_N_4_ via the Au—N bonding, which functioned as efficient electrocatalyst for both OER and ORR.[qv: 66b] This SAC exhibited an outstanding OER activity that was close to the state‐of‐art RuO_2_ commercial catalyst, and the AuN*_x_* species was regarded as the active site for OER process. Park and co‐workers loaded Ni atoms on the g‐C_3_N_4_,^49^ but the OER performance was much worse the above‐mentioned Ni SACs on graphene, which was believed to result from the poor conductivity the carbon nitride support. To improve the performance of Ni/g‐C_3_N_4_ SAC, Liu et al. prepared a dual‐metal (Fe, Ni) SAC on carbon nanotube supported g‐C_3_N_4_, where the isolated metal atoms were bonded with N atoms on carbon nitride.^114^ The codoping of Ni and Fe induced a synergic effect, providing the catalyst with exceptional OER performance (low overpotential of ≈326 mV at 10 mA cm^−2^ and small Tafel slope of 67 mV dec^−1^) which even outperformed the benchmark commercial IrO_2_/C catalyst.

To sum up, similar to HER, OER processes favor the SACs@2D nanoplatform. Well‐defined active centers, uniform structures, controllable bandgaps by tuning the metal loading or metal species, high‐loading metal atoms, and cooperation of metal, and supports lead to highly efficient OER catalysts.

*Other Reactions*: Apart from the most studied applications discussed above, SACs@2D materials were also employed for catalyzing other energy‐related applications. For example, Sun and co‐workers utilized the graphene immobilized Pt SACs for electrocatalytic methanol oxidation, which showed 10 times better activity than benchmark Pt/C catalyst and exhibited great potential for direct methanol fuel cell application.^36^ In addition, Cui et al. anchored isolated Co atoms on N‐doped graphene, which exhibited excellent activity and stability for the inter‐conversion of the redox couple I^−^/I_3_^−^ and is promising for dye‐sensitized solar cells applications. These areas have been intensively explored over metal NPs and more SACs are worth trying in these energy‐related reactions to improve the situation of energy storage and consumption.

### 5.3 Application of SAC on 2D Materials for Solving Environmental Issues

#### 5.3.1 CO_2_ Fixation

The concentration of CO_2_ in the atmosphere keeps increasing because of the high emission speed and decreasing carbon sinks, causing serious consequences including global warming, ocean acidification, and carbon fertilization problems in climate and environment has been caused by the excessive emission of CO_2_. Thus, efficient transformation of CO_2_ to environmentally friendly substances or value‐added chemicals is a hot research topic. Due to the amazing properties of 2D material‐supported metal SACs, applying them to CO_2_ fixation has been explored in different directions.

**Figure 23:**
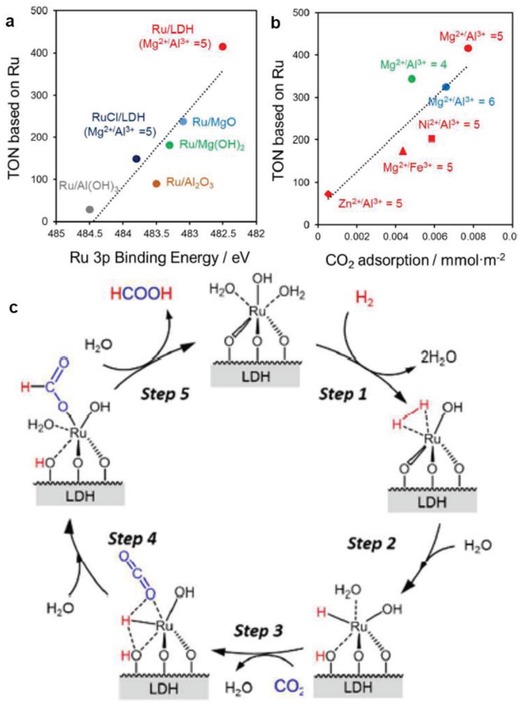
a) Relationship between the TON for CO_2_ hydrogenation on Ru‐based catalysts, and Ru 3p binding energy as determined by XPS spectroscopy. b) Relationship between the TON for CO_2_ hydrogenation based on Ru and CO_2_ adsorption for various LDH supported Ru SACs. c) Possible reaction pathway for CO_2_ hydrogenation to produce formic acid over Ru SAC. Reproduced with permission.^79^ Copyright 2017, American Chemical Society.

*Thermocatalytic CO_2_ Reduction*: Mori et al. synthesized a Ru SAC on MgAl‐LDHs (Mg^2+^/Al^3+^ = 5) through a simple wet‐impregnation method, and applied the SACs to catalyze the hydrogenation of CO_2_ to formic acid.^79^ Under the identical condition, this Ru SAC performed the best among all the tested catalysts (Ru/LDH, LDH, Ru/MgO, Ru/Mg(OH)_2_, Ru/Al_2_O_3_, Ru/Al(OH)_3_, RuCl/LDH, Rh/LDH, Ir/LDH, and RuCl_3_ solution) with the highest TOF of ≈19 h^−1^, which also surpasses many reported efficient catalysts. As can be seen in **Figure** 23a, the catalytic performance has close relationship with the binding energy of Ru 3p electrons, with higher rates obtained Ru species with higher charges. LDHs with different metal pairs were employed for stabilizing Ru atoms, and the MgAl LDH (Mg^2+^/Al^3+^ = 5) was found to the most active. Figure 23b demonstrates that the reaction rate sequence followed that of CO_2_ adsorption amount caused by different basicity of LDHs. A possible reaction pathway based on the above results was proposed in Figure 23c. Similar to the hydrogenation on many oxides supported metal SACs, H_2_ was dissociated in a heterolytic process, which was promoted by the basic ligand. Further, CO_2_ was absorbed on the Ru site, followed by the H^−^ attach and catalyst regeneration. However, this work just regards LDH as an oxide, and the how 2D structure promotes the catalytic cycle was not mentioned. It seems an oxide support could replace LDH after certain modification.

**Figure 24:**
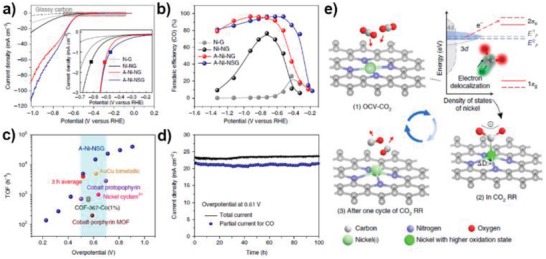
a–d) Electrocatalytic performance of CO_2_ reduction. e) Structural evolution of the active sites during the CO_2_ reduction process. Reproduced with permission.^60^ Copyright 2018, Nature Publishing Group.

*Electrocatalytic CO_2_ Reduction*: As illustrated above, graphitic carbon material‐supported metal SACs have good potential for electrocatalysis. Yang et al. successfully applied N‐doped graphene supported Ni SACs for eletrocatalytic CO_2_ reduction to CO.^60^ The authors prepared two Ni SACs using pyrolysis method with one precursor containing S, and the resulting two SACs are named as A‐Ni‐NG and A‐Ni‐NSG, respectively. Benefiting from the stabilization effect of the 2D support and the electronic interaction among the Ni atoms and support, Ni SACs demonstrated exceptional catalytic performance (**Figure** 24a–d) and high selectivity toward CO (>80%). The A‐Ni‐NSG, which is more active than A‐Ni‐NG, demonstrated an amazing TOF of 14 800 h^−1^ at a mild overpotential of −0.61 V, which is at 10 times of the values for the other catalysts including the previously reported best catalysts. Besides, A‐Ni‐NSG achieved a maximum CO Faradaic efficiency of 97%, at ≈‐0.5 V. This SAC also survived a 100 h run at ‐0.61 V, without detectable decay in activity (22 mA cm^−2^). In situ techniques and theoretical calculation were performed on the simpler A‐Ni‐NG to illustrate the reaction mechanism, and the transformation of catalyst structures were plot in Figure 24e.The result suggested the N‐coordinated Ni^1+^–N_4_ site to be the active center for CO_2_ reduction, and it was the weak bonding between CO and isolated Ni species that facilitate CO desorption, thus inhibiting further reduction. Singly distributed active sites is the unique properties of Interestingly, Wu and co‐workers also investigated eletrocatalytic CO_2_over Ni/N‐doped graphene and found that Ni^2+^ played a key role in transforming CO_2_ to CO. They achieved high selectivity for CO with the CO Faradic efficiency higher than 90% in a wide potential range (‐0.5–0.9 eV).^56^ The highest CO Faradaic efficiency of 99% was obtained at ‐0.81 V with a current density of 28.6 mA cm^−2^. Also based on carbon supported single Ni–N*_x_* site, Wang's group developed another Ni SAC whose Faradaic efficiency reached 95% for CO at −0.62 V with a current density of 11 mA cm^−2^.^115^ Tour and co‐workers synthesized a series of Fe SACs on N‐doped graphene, which showed controllable activity for eletroctalytic CO_2_ reduction by adjusting the pyrolysis temperature.^59^ However, Fe SACs performed worse than the above mentioned Ni SACs, as the highest Faradaic efficiency for CO was merely 80% at −0.6 V with a current density of 2 mA cm^−2^. Loading Nb atoms onto MoS_2_, Salehi‐Khojin and co‐workers obtained an Nb SAC, which also showed good performance for converting CO_2_ to CO.^116^ This Nb SAC prominently enhanced the catalytic performance of MoS_2_ and its highest Faradaic efficiency for CO was found to be 82% at −0.8 V, at which potential the current density reached a high value of237 mA cm^−2^. Theoretical studies indicated that releasing the formed CO from MoS_2_ requires surpassing a high energy barrier, despite the easy formation of CO in it. After incorporating Nb atoms on MoS_2_, the barrier decreased due to the modulation of electronic density of edge Mo species which resulting in weakening the interaction between CO and Mo atoms. Moreover, it was found that positions with Nb doping on the support significantly improves the desorption behavior of CO.

Despite the encouraging results have been obtained in CO_2_ reduction, the threatening of CO must not be ignored. CO is a poisonous gas which does great hard our heath and also destroy a catalyst by strong adsorption on active sites. Therefore, reducing CO_2_ to C—H compounds is more admirable.

*Photocatalytic CO_2_ Reduction*: Metal SACs on g‐C_3_N_4_have showed good performance in photocatalytic water splitting reaction, and the application of them to more photocatalytic reactions has been explored, CO_2_ reduction is a promising reaction for environmental and energy applications. Gao et al. employed theoretical calculations to study g‐C_3_N_4_‐supported Pd and Pt SACs for photocatalytic CO_2_ reduction.^117^ As suggested by the results, red‐shift of the optical absorption spectra toward visible light range was observed after the incorporation of the metal atoms onto carbon nitride support, which boosts the application of g‐C_3_N_4_ supported SACs for photocatalytic CO_2_ reduction using visible light. Apart from noble metal atoms, transition metals may also tune the electronic structure of g‐C_3_N_4_. Experimentally, Huang et al. employed g‐C_3_N_4_ supported cobalt SAC to achieve the selective reduction of CO_2_ to CO under visible light irradiation.^77^ Over the Co SAC, a TON of larger than 200 was achieved for CO formation under visible‐light irradiation. During the preparation process, CoO*_x_* NPs would form at higher Co loading, which was proved inactive for CO_2_ hydrogenation. Fortunately, such oxide did not influence the selectivity toward CO. The locations of single Co atoms and nature of the catalytic performance was investigated via XAS together with a few control samples.

Apart from carbon nitride‐supported metal SACs, Xiong and co‐workers employed 2D oxidized graphene supported Co SACs photocatalytic CO_2_ reduction.^118^ Under visible‐light irradiation (>420 nm), this SAC achieved a high TON value of 678 after modification with [Ru(bpy)_3_]Cl_2_, and the TOF was estimated to be 3.77 min^−1^, surpassing the previously reported state‐of‐art photocatalysts for CO_2_ reduction. This work utilized a homogeneous photosensitizer for enhancing large absorption coefficient for visible light and the Ru complex also promoted the reaction via electron transfer.

Photocatalytic CO_2_ reduction is promising due to the clean and energy‐friendly process. Although quite a few theoretical studies have proved it is approachable over various SACs@2D materials, experimental achievements are yet limited.

#### 5.3.2 Photocatalytic Degradation of Organic Pollutants

Photocatalytic degradation of the organic pollutants in water is an effective solution to the serious water pollution problem, and has received increasing research interests. Effective photocatalysts are thus highly demanded for dealing with the pollutants. Metal SACs on g‐C_3_N_4_ have demonstrated exceptional performance in this application.

Through a pyrolysis process, Guo and co‐workers synthesized a Fe/g‐C_3_N_4_ SAC with the Fe loading reaching up to 18.2 wt%.^119^ This Fe SAC showed exceptional performance in inducing the degradation of series model organic pollutants including methylene blue, methyl orange, rhodamine B, and phenol via the Fenton reaction. The authors correlated the catalytic Fenton performance with the number and dispersion of the FeN*_x_*, i.e., more sites with higher dispersion yield better activity. In neutral media, the most active Fe SAC realized complete removal of all model pollutants within only 15 min, exhibiting encouraging performance. Further investigation confirmed that the isolated Fe‐N*_x_* sites functioned as the active centers, catalyzing the generation of reactive oxygen species (HO∙) from H_2_O_2_.

**Figure 25:**
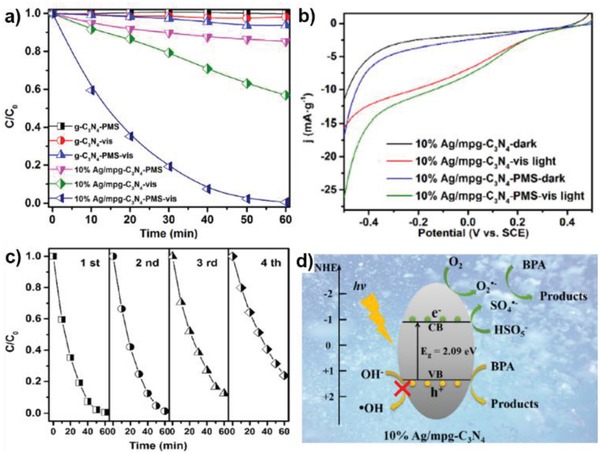
Catalytic performance and proposed reaction mechanism for BPA degradation. a) BPA degradation by activation of PMS. b) LSV curves in the presence or absence of 1.0 mM PMS. c) Cycling test of 10% Ag/mpg‐C_3_N_4_/PMS for the degradation of BPA. d) The possible photocatalytic mechanism in Ag/mpg‐C_3_N_4_/PMS/vis system. Reproduced with permission.^120^ Copyright 2017, Elsevier.

To broaden the photocatalytic applications in pollutants removal, Ag SACs on carbon nitride were also investigated for dealing with the organic pollutants.^120^, ^121^ Zhu and co‐workers applied Ag/mpg‐C_3_N_4_) to catalyze the degradation of bisphenol A (BPA) in presence of an additional oxidant, peroxymonosulfate (PMS).^120^ As shown in **Figure** 25a, PMS itself showed poor activity for BPA degradation, whereas the combination of PMS and Ag SAC dramatically enhanced the performance of Ag SAC. To elucidate the function of PMS in BPA degradation, LSV curves were recorded in Figure 25b, and the drastic difference of current in dark and under irradiation for Ag/mpg‐C_3_N_4_‐PMSsuggested that strong interaction between Ag SAC and PMS upon photoirradiation. This proved that PMS functioned as the electron acceptor and was which receives the photogenerated electrons from Ag SAC to form sulfate radicals. The introduction of Ag atoms benefits the SAC in harvesting more visible light due to the SPR, while the presence of PMA effectively boosts electron–holes separation. Besides, Wang et al. employed Ag/C_3_N_4_ for the visible light‐driven degradation of sulfamethazine in presence of PMS, which also showed outstanding activity.^121^

This field of application is important for environment protection and focuses more on application than fundamental understanding, thus well‐defined SACs@2D materials do not receive much attention. Another factor is that the bandgap for the most used 2D materials is not as good for photocatalysis as 3D supports (TiO_2_, for example).

*Other Reactions*: CO oxidation is another important reaction for eliminating the vehicle exhaust. However, most of the SACs that have been synthesized are based on 3D oxides supports. The main reason is that SACs@3D oxides have better chance to survive high temperatures by incorporating into the support skeleton under the working conditions for dealing with the exhaust in vehicles. Even though low‐temperature CO oxidation has been accomplished over various SACs, high temperature durable SACs are still in demand. Harnessing the wisdom employed in the work conducted by Yan and co‐workers, the reaction The majority of studies on applying 2D material‐supported metal SACs are still based on DFT calculations.^5^, ^122^ Although Pd/TiO_2_ nanosheet and Pt/CuO film were employed for catalyzing CO oxidation,^123^ the performance did not surpass the SACs on 3D supports. Harnessing the wisdom employed in the work conducted by Yan and co‐workers, the reaction activity is tuned by adjusting the redox potential of the supports, 2D supports have advantages in altering their properties over 3D supports, which sheds light on the promising application of SACs@2D for CO removal.[qv: 88c] Thus, more catalytic attempts of 2D material‐supported metal SACs are required for dealing with vehicle exhaust (CO and NO*_x_*).

## 6 Conclusion

### 6.1 Summary

2D materials and SAC are both new frontiers in heterogeneous catalysis, whose combination will help create uniformly precise geometric structure and unique charge state endowing 2D material‐supported metal SACs unlimited potential in catalytic applications. In recent years, increasing research interest has been attracted to this emerging frontier field, and 2D material‐supported metal SACs are employed for catalyzing quite a few reactions related to value‐added chemical production, energy conversion, and environment protection, making great contributions the world. The relatively uniform structures of 2D materials also benefit fundamental understanding of the chemical processes via experimental techniques and theoretical calculations. This review summarizes the recent synthetic strategies for metal SACs@2D supports, followed by discussing the current application of these catalysts in producing value‐added chemicals, energy and environmental protection related reactions. Compared to 3D supports, 2D supports make it possible to realize well‐defined and well‐distributed catalytically active sites, which are ideal platforms for performing fundamental studies and catalyzing reactions with high selectivity. The tunable electron density, coordination structures, and bandgaps of 2D supports with heteroatom dopant, making the combination of metal atoms and 2D supports exceptionally promising for controllable design of ideal SACs with specific targets. Although satisfactory results have been achieved, more efforts are required to make SACs@2D materials stand out as an excellent candidate in industry.

### 6.2 Outlook

- In this review article, LDH, carbon materials and MoS_2_ are the most discussed 2D supports. Apart from these, many emerging 2D materials such as MXene, Xenes, BN, and so on also showed promising properties for stabilizing single metal atoms. Researcher have already employed DFT calculations to prove the feasibility of confining metal atoms onto these 2D supports, but experimental verification and practical application tests are still required. MXene‐supported Pt SAC has been obtained via an electrochemical deposition method and showed good performance for HER reaction. As a promising 2D material, MXenes with more than 30 compositions have been reported, which provides a series of excellent 2D supports for stabilizing metal atoms.^124^ Furthermore, our group has made quite a few achievements in 2D Xenes such as black phosphorus (BP), which is now a research frontier in 2D materials.[qv: 9,12b,125] 2D BP shows excellent physical and chemical properties such as direct and layer‐dependent bandgap and high mobility of carrier, and has proved good photothermal and photochemical capability. Thus, it can be anticipated that the combination of 2D BP with metal single atoms will open a new window for photocatalysis and photothermal catalysis. Theoretical studies already pointed out the great potential of BP supported Fe SAC for NH_3_ synthesis, which is a highly important reaction in industry. To sum up, new routes and new 2D materials are worth exploring for constructing efficient and useful metal SACs.
- Thanks to the uniformly well‐defined structures, it is feasible to perform theory‐guided synthesis of metal SACs@2D supports. Theoretical calculations are performed first for a given support to check their ability to stabilize metal atoms and corresponding catalytic activities. As the theoretical result is more convincing over uniform 2D materials than heterogeneous 3D materials, the combination of metal and 2D supports during SAC synthesis will become much easier with the guidance of theoretical studies and the resulting SACs are probably effective for the desired reaction. However, theoretical calculations over such exciting platforms should be more sophisticated to mimic the real case, which might provide more insightful information. As to the experimental evidences, in situ/operando techniques such as in situ XAS, in situ XPS and in situ IR together with ex situ techniques must be adopted, to consolidate the understanding of how catalysis occurs.
- At current stage, we have to admit that the applications of 2D material‐supported SACs are quite limited. For chemical production, the chemical scope is much narrower than that of 3D material supported metal SACs. In environmental are, metal SACs on 3D supports can efficiently catalyze the reduction of NO*_x_* and various choice for low temperature CO oxidation, while 2D material‐supported SACs only focused on CO_2_ reduction and organic decomposition. Although SACs@2D materials are widely used for catalyzing HER process, they have much fewer results on OER, which is more challenging. More importantly, despite the fact that SACs@2D materials outperform SACs@3D materials in mechanistic studies, SACs@2D material requires more exploration to demonstrate that they are irreplaceable by 3D supported ones in applications. SACs@2D materials will eventually serve as industrial catalysts, not just model ones. Therefore, future studies should enrich the research scope of the three main areas discussed in this review.
- Moreover, 2D material‐supported SACs showed encouraging performance in electrocatalysis and photocatalysis, which provided a good platform for antibacterial and antitumor applications. For example, 2D material‐supported metal NPs and organometallic complex have been applied for photodynamic therapy and H_2_ therapy, as the metal species can catalyze Fenton reaction or water splitting reaction to generate activated oxygen species and H_2_, respectively. Considering the low concentration of O_2_ in cancer cells, photocatalytic oxidation of water under NIR to continuously supply O_2_ is an ideal solution. Thus, preparing efficient OER metal SACs on biocompatible and biodegradable 2D materials provides a good directly for applying the catalysts to medical area.^126^ In addition, SACs have attracted the attention of researchers in nanozyme, which is also very promising.^127^
- Table 1 summarizes quite a few approaches for synthesizing metal SACs over 2D supports, but how to realize scalable and controllable synthesis is still under exploration. Pyrolysis method can achieve high metal loading, but it is not easy to precisely control the number of active sites and the resultant catalysts usually have heterogeneous structures. Creating defects and introducing heteroatoms to the 2D materials damage the original structures, so whether the unique advantages are reserved remains unclear. ALD method can achieve controllable metal loading, but the stability under harsh condition is under debate. Furthermore, the expensive cost hinders its scalable application. Wet‐chemistry method can solve the problem of scalable production, but suffer from controllable issue and the precise position of active sites may not follow a homogeneous distribution. Thus, developing facile, controllable, scalable, and economic approaches for 2D material‐supported metal SAC synthesis is an urgent problem for this field.

## Conflict of Interest

The authors declare no conflict of interest.
